# Top-down medial prefrontal cortex-to-hypothalamic paraventricular nucleus circuit regulates social avoidance and conditioned fear in male mice

**DOI:** 10.1038/s41421-026-00893-6

**Published:** 2026-06-09

**Authors:** Yu Wang, Xiang-Yu Pan, Bo Wu, Dan-Yang Li, Shuo-Wen Wang, Xin-Ya Qin, Qing-Hong Shan, Peng Chen, Pu Hu, Hao Wang, Rong-Yu Liu, Hui Gong, Jiang-Ning Zhou

**Affiliations:** 1 Institute of Brain Science, The First Affiliated Hospital of Anhui Medical University, Hefei, Anhui China; 2 Anhui Provincial Key Laboratory for Brain Bank Construction and Resource Utilization, Anhui Medical University, Hefei, Anhui China; 3 Anhui Province Key Laboratory of Biomedical Imaging and Intelligent Processing, Institute of Artificial Intelligence, Hefei Comprehensive National Science Center, Hefei, Anhui China; 4 National Engineering Laboratory for Brain-inspired Intelligence Technology and Application, MoE Key Laboratory of Brain-inspired Intelligent Perception and Cognition, School of Information Science and Technology, University of Science and Technology of China, Hefei, Anhui China; 5 Anhui Geriatrics Institute, the First Affiliated Hospital of Anhui Medical University, Hefei, Anhui China; 6 HUST-Suzhou Institute for Brainsmatics, JITRI, Suzhou, Jiangsu China; 7 Division of Life Sciences and Medicine, University of Science and Technology of China, Hefei, Anhui China

**Keywords:** Mechanisms of disease, Calcium signalling

## Abstract

Compromised cortical inhibition during threat processing contributes to individual vulnerability to stress-related psychiatric disorders. However, the precise underlying neurobiological circuits remain elusive. Here, by combining monosynaptic viral tracing, electrophysiology, in vivo calcium imaging, and functional manipulations in mice, we elucidated a functionally specialized monosynaptic pathway originating from glutamatergic pyramidal neurons in the ventromedial prefrontal cortex (vmPFC^Glu^) to corticotropin-releasing hormone (CRH)-expressing neurons in the paraventricular nucleus of the hypothalamus (PVN^CRH^). We found that activating medial prefrontal cortex (mPFC)-driven CRH “pacemaker” cells propagated calcium signals within the local CRH network of the PVN. Hyperactivity of this vmPFC^Glu^-PVN^CRH^ circuit promoted persistent social avoidance, consolidated threat memory, facilitated auditory-cued fear acquisition, and impaired extinction. Conversely, inhibition of this circuit selectively reduced social stress‑induced avoidance. Our findings define a novel top-down circuit that specifically controls psychosocial stress responses and amplifies susceptibility to conditioned fear.

## Introduction

Exposure and a dysregulated response to severe psychological stress are associated with the development of neuropsychiatric disorders such as post-traumatic stress disorder (PTSD)^1–3^. PTSD is increasingly being recognized as a disorder of neural circuits^3–6^ and is characterized by persistent fear memory re-experience and impaired fear extinction — symptoms rooted in dysregulated interactions between cortical and subcortical stress circuits^4,6–16^. Emerging evidence points toward potential aberrations in limbic circuit control underlying PTSD^6^. The ventromedial prefrontal cortex (vmPFC) serves as a crucial node within this network, exerting top-down inhibitory control over amygdala-dependent fear responses and promoting extinction learning^4,8,17^. Nevertheless, in individuals with PTSD, hypoactivity of the medial prefrontal cortex (mPFC) is correlated with exaggerated fear recall and failed extinction, suggesting a breakdown of cortical control over limbic hyperactivity^18^. On the other hand, multiple studies have reported PTSD-associated hypersensitivity to hypothalamic–pituitary–adrenal (HPA) feedback^19–21^. This hypersensitivity is thought to be related to chronic hyperactivity of corticotropin-releasing hormone (CRH) neurons in the hypothalamic paraventricular nucleus (PVN). However, data on baseline levels of adrenocorticotropic hormone and cortisol in individuals with PTSD are somewhat variable^4^. The finding of low cortisol levels in PTSD patients is inconsistent with the hypothesis that stress-induced glucocorticoid elevation impairs mPFC function and increases the effects of glucocorticoids on the amygdala^7^. While the existing evidence confirms that the mPFC is pivotal in PTSD-associated fear regulation and HPA axis dysregulation, its precise neuroendocrine interaction with hypothalamic circuits and mechanistic contributions to threat/fear-related behavioral phenotypes remain unresolved.

Recent studies have begun to elucidate the role of prefrontal–hypothalamic circuits in stress adaptation^22,23^. The mPFC plays a crucial role in regulating the HPA axis and is associated with psychological stimuli-induced adrenocorticotropic hormone (ACTH) or cortisol levels in human and animal studies^24–28^ by generally inhibiting stress-induced activation of the HPA axis. The prevailing model posits that indirect mPFC pathways — likely mediated by intermediary nuclei such as the bed nucleus of the stria terminalis (BNST) — attenuate HPA axis activation via inhibitory GABAergic signaling to hypothalamic paraventricular neurons^29,30^. Recent advancements in neural techniques have revealed a growing array of structural and functional connections among neuron types with distinct characteristics across various brain regions, including the mPFC^31–34^, leading to increased controversy regarding the anatomical and functional relationships between the mPFC and hypothalamus/PVN. For example, emerging evidence suggests that direct projections from the mPFC to the PVN may rapidly amplify stress signals^35^ or regulate the endocrine response to stress^36–38^. Moreover, the mPFC-dorsal hypothalamus pathway participates in the top-down modulation of psychological stress responses^23^. This dichotomy raises critical questions: Does hyperactivity in the mPFC-PVN^CRH^ pathway override the canonical fear-inhibitory function of the vmPFC, and could such dysregulation contribute to PTSD vulnerability? Here, we focus on the mPFC-PVN^CRH^ circuit in an attempt to provide a precise anatomical and functional analysis of its role in regulating threat/fear-related responses in mice.

In this study, we integrate monosynaptic viral tracing, electrophysiological recordings, calcium imaging, and chemogenetics in murine models to elucidate the functional role of a direct vmPFC^Glu^-PVN^CRH^ circuit in the pathogenesis of stress-related psychopathology. We demonstrate that this pathway (1) exhibits stimulus-specific activation during psychosocial stress, (2) drives persistent social avoidance and threat memory consolidation when hyperactive, and (3) promotes fear learning and impairs fear extinction. Crucially, while activation of the vmPFC^Glu^-PVN^CRH^ circuit mimics PTSD-like phenotypes, its inhibition selectively alleviates social stress-induced avoidance. This dissociation underscores its specialized role in encoding threat salience rather than generalized fear processing. These results identify a novel top-down circuit that functionally couples cortical fear regulation with hypothalamic stress effector systems and suggest that hyperactivation of this circuit may constitute a neural substrate that heightens individual vulnerability to trauma-related psychopathology, potentially predisposing individuals to PTSD-like phenotypes.

## Results

### Pyramidal cells in the vmPFC provide monosynaptic innervation to CRH neurons in the PVN

**Fig. 1:**
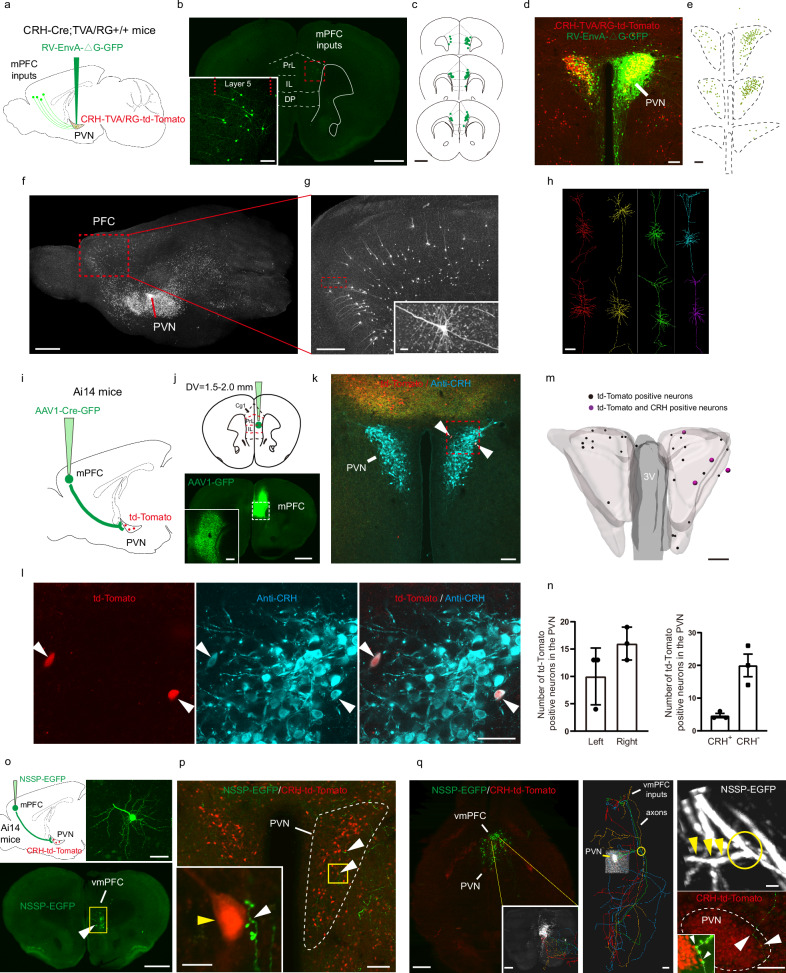
Monosynaptic circuit tracing reveals vmPFC-PVN^CRH^ connectivity. **a** Strategy for tracing the monosynaptic RV of mPFC inputs to PVN^CRH^ neurons. **b** Distribution of RV-labeled inputs from mPFC subregions; the inset shows magnified inputs. Scale bars: 1 mm; inset, 100 μm; *n* = 6 mice. **c** Schematic of the input distribution across mPFC coronal planes. Each green dot represents an input soma. Scale bar: 1 mm. **d** PVN expression: CRH neurons (td-Tomato), RV-labeled neurons (EGFP), and starter neurons (merged). Scale bar: 100 μm. **e** Distribution of starter neurons within the PVN across planes; the dotted line indicates the PVN boundary. Scale bar: 100 μm. **f** 3D whole-brain rendering of mPFC input neurons. Scale bar: 1 mm. **g** Enlarged view showing the morphological details of single-input neurons; the inset depicts the soma, dendrites, and axon of a pyramidal neuron. Scale bars: 200 μm; inset, 20 μm. **h** Reconstructed mPFC input neurons exhibit a pyramidal morphology. Scale bar: 200 μm. **i** Anterograde trans-monosynaptic AAV1 tracing strategy from the mPFC to the PVN. **j** AAV1 injection site (schematic) and GFP expression in the mPFC; the inset shows magnified neurons. Scale bars: 1 mm; inset, 200 μm. **k**, **l** AAV1-labeled CRH neurons in the PVN (merged, arrowheads); (**l**) boxed region in (**k)**. Scale bars: 100 μm (**k**); 50 μm (**l**). **m** 3D schematic of AAV1-labeled CRH and non-CRH neurons in the PVN. Scale bar: 100 μm. **n** Quantification of postsynaptic neurons: left- vs right-PVN (left), CRH vs non-CRH neurons (right). *n* = 3 mice. **o** Single-neuron labeling in the vmPFC; the inset highlights a GFP-labeled pyramidal neuron (arrowhead). Scale bars: overview, 1 mm; inset, 50 μm. **p** Long-range axonal terminals in the PVN; the inset shows axons passing through a CRH soma (yellow arrowhead) and forming synaptic structures (white arrowhead). Scale bars: 100 μm; inset, 10 μm. **q** 3D whole-brain view of GFP-labeled mPFC neurons (left), reconstructed pyramidal cells with axonal branches toward the PVN (middle), a branch extending to the PVN (top right, yellow circle/arrowheads), and traced terminals adjacent to CRH neurons (bottom right, inset: magnified view showing axon terminals in close apposition to CRH neuron somata, white arrowheads). Scale bars: left, 300 μm; inset, 500 μm; middle, 500 μm; top right, 1 μm; bottom right, 100 μm.

To identify direct synaptic connections from the mPFC to PVN^CRH^ neurons, we first mapped the sources of inputs to these neurons using rabies virus (RV) monosynaptic retrograde tracing in CRH-Cre;TVA/G^loxP/loxP^ mice (CRH^TVA/G+/+^ mice), in which the avian viral receptor TVA and rabies glycoprotein G were fused to td-Tomato and expressed specifically in CRH-expressing cells. RV-EnvA-ΔG-EGFP was injected into the PVN of the CRH^TVA/G+/+^ mice (Fig. 1a). Seven days later, the brain sections were processed, revealing GFP-expressing direct presynaptic inputs from the mPFC (Fig. 1b). Most of these input cell bodies were located in layer 5 of the prelimbic (PrL) and infralimbic (IL) subregions (collectively referred to as the vmPFC^39,40^) (Fig. 1b, c). To ensure that starter neurons were confined to the PVN, only samples with minimal viral spread were analyzed (Fig. 1d, e). We tested RV injection volumes of 20, 50, or 100 nL into the PVN to examine the effect of viral dose on input labeling. Injections of 20 or 50 nL effectively restricted starter spillover while labeling a comparable number of inputs (Supplementary Fig. S1). We then used fluorescence micro-optical sectioning tomography (fMOST) to acquire high-resolution whole-brain morphological images of the labeled inputs (Fig. 1f, g). Subsequent reconstruction of individual mPFC neurons revealed their characteristic pyramidal morphology (Fig. 1h).

To validate the projection from vmPFC neurons to PVN^CRH^ neurons, we employed an anterograde monosynaptic tracing strategy (Fig. 1i). AAV1-Cre-GFP was injected into the PrL/IL subregions of Ai14 transgenic mice (Fig. 1j; Supplementary Fig. S2a), followed by immunostaining for CRH in the PVN. This revealed td-Tomato^+^ neurons in the PVN that received mPFC input, with a subset of these neurons exhibiting colocalized CRH immunoreactivity (Fig. 1k, l; white arrows; Supplementary Fig. S2b). We reconstructed the entire PVN to map the spatial distribution of these neurons (Fig. 1m) and quantified the proportion of CRH^+^ neurons among all td-Tomato^+^ neurons receiving mPFC innervation (Fig. 1n). The specificity of viral expression in the mPFC, with no leakage into subcortical regions such as the septum (which could also project to PVN^CRH^ neurons), is shown in Supplementary Fig. S2c and d.

We next used sparse labeling, whole‑brain imaging, and single-cell reconstruction to further delineate this circuit. AAV-NSSP-EGFP was injected into the vmPFC or dorsomedial prefrontal cortex (dmPFC) of CRH-Cre;Ai14 mice (Fig. 1o, top left). Four weeks later, whole-brain imaging at single-cell resolution revealed sparsely and fully labeled pyramidal neurons in the vmPFC (Fig. 1o, bottom and top right). Their axons extended into the PVN and formed presynaptic boutons around CRH neuron somata (Fig. 1p). Whole‑brain axon tracing and neuronal reconstruction (Fig. 1q, left) revealed that lateral branches of the long projections from vmPFC pyramidal neurons entered the PVN (Fig. 1q, middle and top right; the yellow circles indicate axonal branch points) and formed appositions with CRH neurons (Fig. 1q, bottom right). In contrast, no axons or branches from dmPFC neurons were observed in the PVN or other hypothalamic regions (Supplementary Fig. S3a–c), whereas contralateral projections in the mPFC and ipsilateral projections from the dmPFC to the striatum were detected (Supplementary Fig. S3d–g).

Together, using viral tracing and neuronal reconstruction, we demonstrate the existence of monosynaptic connections from vmPFC pyramidal cells to PVN^CRH^ neurons (vmPFC-PVN^CRH^).

### vmPFC projection neurons control PVN^CRH^ neurons by initiating pacemaker cells in the PVN

**Fig. 2:**
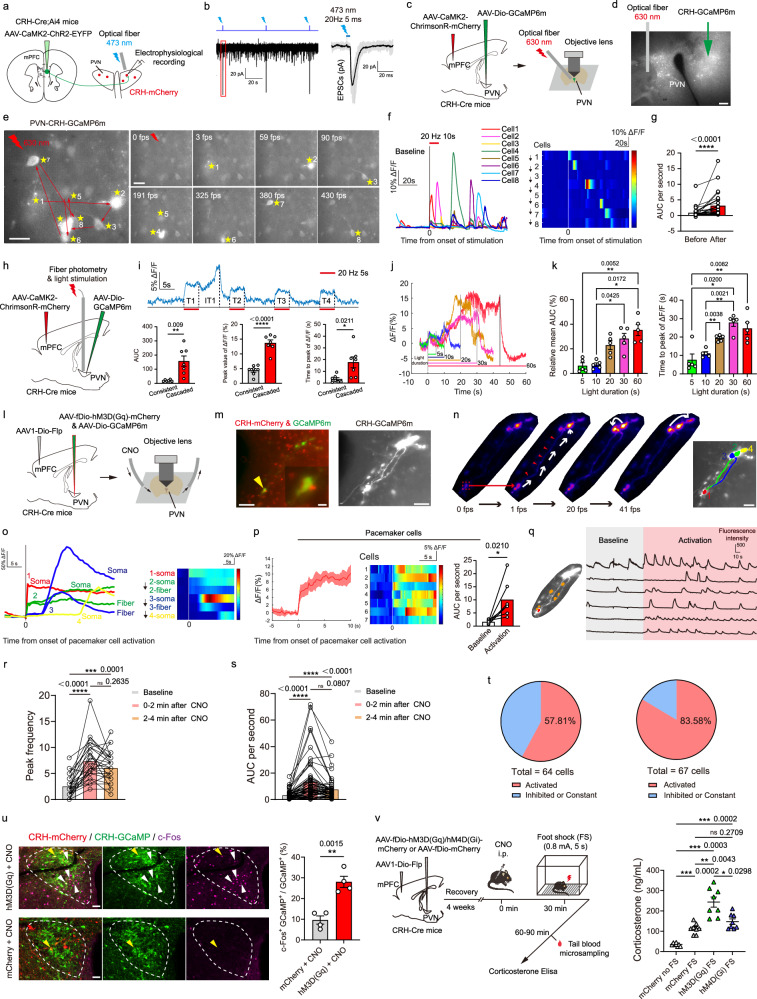
Pacemaker cells mediate the excitatory effect of the vmPFC on PVN^CRH^ neurons. **a** Strategy to record mPFC-evoked EPSCs in PVN^CRH^ neurons. **b** EPSC traces in PVN^CRH^ neurons optogenetically evoked by mPFC inputs. *n* = 5 neurons from 3 mice. **c** Strategy for optogenetic stimulation of mPFC-derived terminals and calcium imaging of PVN^CRH^ neurons in slices. **d** Representative image of combined optogenetic stimulation and calcium imaging in a slice. Scale bar: 100 μm. **e** Calcium dynamics poststimulation (representative time series). Symbols indicate responsive neurons and their activation sequence. Scale bars: 20 μm. **f** Representative traces and heatmaps of calcium transients from the cells shown in **e**. **g** AUC per second of calcium transients pre- and post-stimulation. *n* = 30 cells from slices of 3 mice. **h** Strategy for in vivo terminal photostimulation and fiber photometry recording. **i** Representative calcium trace (top) and analysis of two response patterns (lower). *n* = 7 pulses from 4 mice per group. **j** Representative traces evoked by light pulses of different durations. The gray lines indicate light onset. **k** Differences in relative AUC (% of total AUC) and time-to-peak (ΔF/F%) with different light durations. *n* = 5 mice. **l** Strategy for chemogenetic activation and calcium imaging with CNO in slices. **m** CRH neurons expressing mCherry or GCaMP6m in the PVN. Left: arrowhead indicates a coexpressing pacemaker cell (inset shows enlargement). Scale bars: 50 μm; inset, 10 μm. Right: pacemaker cell and surrounding neurons. Scale bar: 50 μm. **n** Fire lookup-table (LUT) time series images of CNO-induced calcium dynamics in a pacemaker cell and local propagation. Red box/arrow: pacemaker cell and calcium transient; white arrows: propagation; red arrowheads: connecting fibers. Right: color-coded structures of the pacemaker cell (soma 1, red) and other involved neurons (soma 2 and fiber, green; soma 3 and fiber, blue; soma 3, yellow). Scale bar: 50 μm. **o** Individual traces, heatmaps and propagation order for the pacemaker cell marked in **n** in response to CNO. **p** Average traces, heatmaps, and AUCs per second for pacemaker cells post-CNO. *n* = 7 cells from 3 mice. **q** Representative marked cells (orange) and calcium traces from surrounding CRH neurons during baseline and after CNO treatment. **r** Peak frequency in PVN neurons at baseline and 0–2 or 2–4 min post-CNO. *n* = 29 cells from 3 mice. **s** AUC per second in activated PVN neurons at baseline and 0–2 or 2–4 min post-CNO. *n* = 54 cells from 3 mice. **t** Differences in calcium activity and the proportion of activated neurons under the strategies in **c** (left) and **l** (right). **u** Left: c-Fos expression in hM3D(Gq)-mCherry^+^ and CRH-GCaMP^+^ cells post-CNO vs control. Yellow arrowheads: c-Fos colocalization with mCherry and CRH-GCaMP; white arrowheads: c-Fos colocalization with CRH-GCaMP only. Scale bars: 50 μm. Right: percentage of c-Fos^+^ CRH-GCaMP^+^ cells among CRH-GCaMP^+^ cells. *n* = 4 mice per group. **v** Strategy for chemogenetic activation or inhibition of vmPFC-projecting CRH neurons before FS and serum corticosterone measurements in the mCherry control (no FS and FS), hM3D(Gq), and hM4D(Gi) groups. *n* = 8 mice per group. ^∗^*P* < 0.05, ^∗∗^*P* < 0.01, ^∗∗∗ ^*P* < 0.001, ^∗∗∗∗^*P* < 0.0001; ns, not significant. The data are presented as the mean ± SEM. See Supplementary Table S1 for statistical details.

The function of the vmPFC-PVN^CRH^ circuit was systematically elucidated through a combination of in vitro and in vivo experiments. First, the CaMK2-ChR2-EYFP virus was transfected into the vmPFC of CRH-Cre;Ai14 mice. The electrophysiological activity of PVN^CRH^ neurons was then recorded in vitro using patch clamping while optogenetically activating the axonal terminals from the vmPFC within the PVN via blue light stimulation (Fig. 2a). Using paired-pulse laser stimulation (473 nm, 5 ms), we recorded excitatory postsynaptic currents (EPSCs), with the amplitude of optically elicited EPSCs being 86.77 ± 16.33 pA (Fig. 2b). Next, we transfected the CaMK2-ChrimsonR-mCherry virus into the vmPFC of CRH-Cre mice and induced GCaMP6m expression in PVN^CRH^ neurons (Fig. 2c). During calcium activity recording in brain slices, red light stimulation activated the vmPFC axon terminals in the PVN (Fig. 2d), allowing observation of light-induced changes in the average fluorescent calcium signal intensity of PVN^CRH^ neurons. The calcium signal in the CRH neurons was sequentially activated (Fig. 2e–g, from cell 1 to cell 8). Furthermore, in vivo experiments (Fig. 2h) demonstrated that optogenetic activation (630 nm, 20 Hz) of vmPFC CaMK2^+^ neuron terminals in the PVN induced a rapid and consistent increase in the CRH calcium signal following light onset (Fig. 2i, top). Notably, the calcium signal exhibited a specific, cascading increase triggered by the initial light stimulation (Fig. 2i, top, T1, first pulse rising phase). The signal continued to increase even after light cessation during the poststimulation period (Fig. 2i, top, IT1, first interpulse interval). This cascade effect was distinct from that of the calcium transient pattern observed in directly photocoupled systems (Fig. 2i, top, T2–T4), featuring increases in the area under the curve (AUC), peak amplitude, and peak latency (Fig. 2i, bottom). To assess stimulus duration dependence, we compared calcium transients evoked by 5, 10, 20, 30, and 60 s of photostimulation. The calcium signal continuously increased after 5, 10, 20, and 30 s of photostimulation (Fig. 2j, k), typically peaking at the end of the light pulse (Fig. 2j, gray vertical lines for 5/10/20/30 s). However, under 60-s stimulation, the signal peaked and began to decline approximately 45 seconds after light onset, suggesting the induction of a refractory period during prolonged light exposure (Fig. 2j, 60 s gray line). The relative mean AUC and peak latency increased with increasing light exposure across all conditions (Fig. 2k). These results demonstrate that presynaptic vmPFC projections potently activate PVN^CRH^ neurons, eliciting a robust and amplified calcium wave cascade.

To confirm the monosynaptic nature of this regulation, we employed a rigorous methodological approach. AAV1-Dio-Flp was injected into the vmPFC, while AAV-fDio-hM3D(Gq)-mCherry and AAV-Dio-GCaMP6m were coinjected into the PVN of CRH-Cre mice (Fig. 2l). This allowed specific chemogenetic activation of PVN^CRH^ neurons receiving direct innervation from the vmPFC. We recorded calcium signals from both putative “pacemaker” CRH neurons (monosynaptically innervated by the vmPFC; Fig. 2m, left; arrowhead) and adjacent CRH neurons (Fig. 2m, right) before and after Clozapine N-oxide (CNO) administration. Following CNO, CRH pacemaker cells showed altered calcium activity, characterized by increased fluorescence intensity (Fig. 2n, p, red dotted box/arrow). This activity then propagated to distant CRH-expressing cells via local fibers (Fig. 2n, o; in n, red arrowheads indicate connecting fibers, white arrows denote the propagation direction, and the pacemaker as cell 1 with color-coded sequential signal recipients is labeled in the right panel; in o, numbers/colors correspond to the cells or fibers in n, with arrows indicating propagation). Moreover, CRH neurons both near and distant from the pacemaker cell (Fig. 2q, left, orange) exhibited increased calcium activity after CNO, as shown by increased fluorescence intensity or peak frequency compared with baseline (Fig. 2q, right). The mean peak frequency of the calcium signal (Fig. 2r) was significantly elevated during the 0–2- and 2–4-min intervals after CNO compared with baseline, although it decreased in the 2–4-min interval relative to that during the 0–2 min interval. The AUC per second (Fig. 2s) significantly increased during the first 0–2 min after CNO and then decreased during the subsequent 2–4 min. In summary, compared with broad presynaptic terminal activation, this targeted monosynaptic manipulation of CRH pacemaker neurons (Fig. 2l–s) elicited a significantly greater calcium response in PVN^CRH^ neurons (Fig. 2t).

To confirm CRH neuron activation in vivo, we performed immunofluorescence staining for c-Fos on brain slices collected 3 h after intraperitoneal injection of CNO. The results revealed high c-Fos expression in the PVN pacemaker neurons (Fig. 2u, top, yellow arrows). Additionally, significant activation was observed in some nonpacemaker CRH neurons (Fig. 2u, top, white arrows) and non-CRH neurons within the PVN, leading to a marked overall increase in c-Fos levels compared with those in controls (Fig. 2u, statistical plot; Supplementary Fig. S4a). These data indicate that specific CRH neuron populations act as initiation nodes within the vmPFC-PVN^CRH^ circuit, efficiently propagating excitatory signals to other local neurons.

Finally, to evaluate the impact of chemogenetic modulation of the vmPFC-PVN^CRH^ circuit on the HPA axis, we measured serum corticosterone levels in tail vein blood collected from mice under either stress or nonstress conditions following the expression of hM3D(Gq), hM4D(Gi), or control constructs (Fig. 2v; Supplementary Fig. S4b). Chemogenetic activation of this circuit prior to foot-shock (FS) stress increased serum corticosterone levels compared with those in the stressed control group without circuit activation (Fig. 2v, mCherry FS vs hM3D(Gq) FS). Preinhibition of the circuit resulted in no significant difference in poststress corticosterone levels relative to those in the control FS group (Fig. 2v, mCherry FS vs hM4D(Gi) FS). In mice not subjected to stress, chemogenetic activation of the circuit did not significantly alter corticosterone levels (Supplementary Fig. S4b).

Taken together, the results of both in vitro and in vivo experiments demonstrated that a specific subset of PVN^CRH^ neurons functions as a group of pacemaker cells within the vmPFC-PVN^CRH^ circuit. These cells activate downstream neuronal populations and increase the reactivity of the HPA axis specifically under stress conditions.

### vmPFC-PVN^CRH^ circuit dynamics underlie social defeat-induced fear encoding and stress responses

**Fig. 3:**
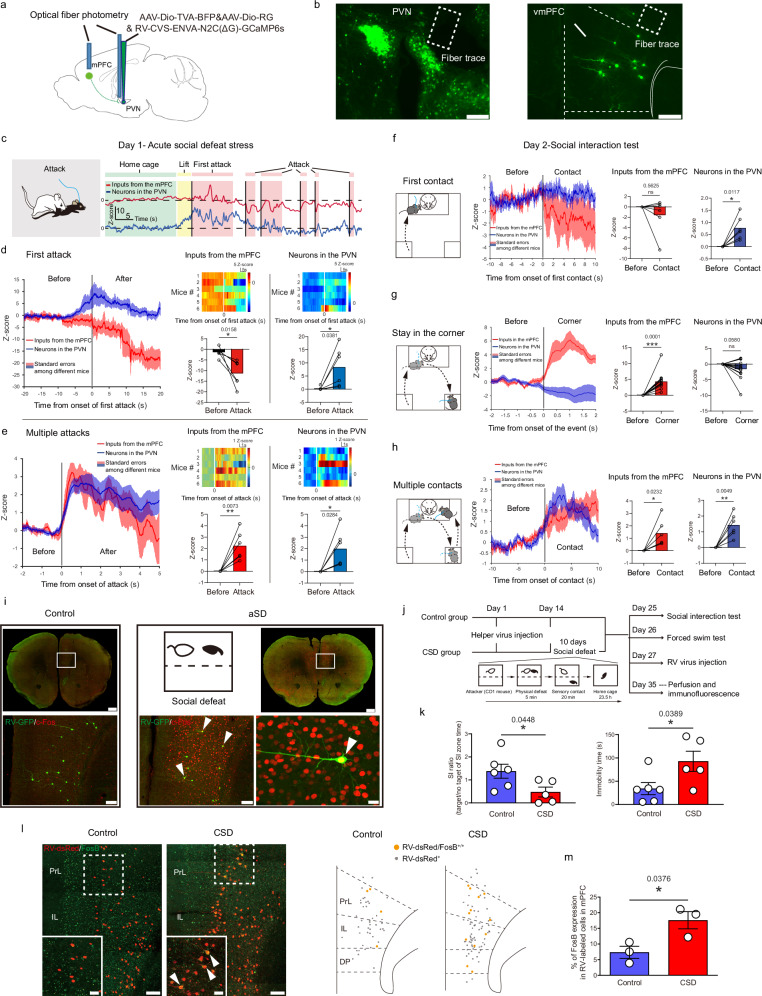
vmPFC input neurons are activated during social psychosocial stress. **a** Strategy for in vivo calcium imaging of vmPFC inputs and PVN neurons using RV-GCaMP6s. **b** Representative images showing optical fiber placement for recording from PVN^CRH^ and vmPFC input neurons. Scale bars: 200 μm. **c** SD paradigm (Day 1) and representative *Z* score traces of calcium signals in vmPFC inputs and PVN neurons across behavioral phases. Pink shading indicates attack periods. **d** Average traces, heatmaps, and peak *Z* scores of calcium signals in inputs and PVN neurons during the first attack (Day 1). *n* = 6 mice. **e** Average traces, heatmaps, and peak *Z* scores during repeated attacks (Day 1). *n* = 6 mice. **f**–**h** Schematics and corresponding calcium signals (average traces and peak *Z* scores) in the inputs and PVN neurons during the social interaction test (Day 2). *n* = 6 mice for first contact (**f**); *n* = 14 events from 6 mice for corner stay (**g**); *n* = 6 mice for repeated contact (**h**). **i** Representative images of c-Fos expression in vmPFC inputs from the control (left) and aSD (right) groups. The bottom panels show magnified views; the arrows indicate c-Fos and RV-GFP colocalization. Scale bars: overview, 500 μm; magnified views, 100 μm (control), 100 μm, and 10 μm (aSD). **j** Viral labeling and behavioral testing timeline under CSD. **k** Social interaction ratio (with CD-1 mouse) and immobility time in the forced swimming test for the control and CSD groups. *n* = 5–6 mice per group. **l** FosB expression in mPFC inputs from control and CSD mice. The arrows indicate colocalization of FosB and RV-dsRed. Schematics show distributions of FosB^+^ input neurons. Scale bars: 100 μm; inserts, 20 μm. **m** Proportion of FosB^+^ input neurons in the mPFC from the control and CSD groups. *n* = 3 mice per group. ^∗^*P* < 0.05, ^∗∗^*P* < 0.01, ^∗∗∗∗^*P* < 0.0001; ns, not significant. The data are presented as the mean ± SEM. See Supplementary Table S1 for statistical details.

Social defeat (SD) stress is a well-established model of psychological stress^23,41^ and has been used to evaluate PTSD-like symptoms^42,43^. Using this paradigm, we first examined changes in the activity of the vmPFC-PVN^CRH^ circuit during psychosocial stress. AAV helper viruses (AAV-Dio-TVA-BFP and AAV-Dio-RG) and RV-CVS-ENVA-N2C(ΔG)-GCaMP6s were injected in the PVN of CRH-Cre mice. Optical fibers were then implanted in both the vmPFC and the PVN to enable simultaneous recording of calcium activity in these regions (Fig. 3a). This approach allowed us to monitor dynamic calcium signaling in PVN neurons and vmPFC inputs (Fig. 3b) throughout the behavioral procedure.

During the first acute social defeat (aSD) session, the initial attack by a CD-1 aggressor mouse triggered distinct changes in circuit dynamics: compared with preattack baseline neurons, vmPFC inputs resulted in decreased *Z* score trajectories of calcium signals, whereas PVN neurons exhibited increases in both global calcium signals and peak amplitudes after attack (Fig. 3c, d). Upon repeated attacks, calcium signals rapidly increased in both vmPFC inputs and PVN neurons immediately after aggression, followed by a gradual decrease that remained above baseline (Fig. 3e). We subsequently performed correlation analysis between mPFC input and PVN neuron signals during attack events. Time-lagged cross-correlation suggested that mPFC input and PVN neuron signals are highly synchronized during attack (mean time lag = −0.12 s) (Supplementary Fig. S5a). These results suggest that vmPFC inputs may encode learned fear memory, whereas PVN neurons likely encode stimulus-evoked fear during the initial defeat experience.

On the second day of the social interaction test (Fig. 3f–h), unlike during passive CD-1 exposure on Day 1, vmPFC inputs displayed a decreasing but not significant trend in the average calcium signal during the first social contact event ( ± 10 s window; Fig. 3f). Notably, PVN neuronal ensembles maintained significantly elevated average *Z* scores after the first CD-1 encounter (Fig. 3f). Interestingly, when mice repeatedly interacted with the CD-1 mouse and then retreated to a corner, the calcium signal in vmPFC inputs increased, whereas no significant change was detected in PVN neurons (Fig. 3g). Across successive social contact trials, both vmPFC inputs and PVN neurons demonstrated progressive increases in the average *Z* scores of calcium signals (Fig. 3h). Cross-correlation analysis revealed that vmPFC input activity increased prior to PVN neuron activity (mean time lag = −1.14 s) (Supplementary Fig. S5b). These findings indicate that vmPFC inputs may retrieve preexisting fear memories, whereas PVN neurons appear to consolidate fear memories during repeated social interactions on Day 2.

Compared with the controls, aSD also induced a significant increase in c-Fos expression in vmPFC inputs, as evidenced by elevated c-Fos levels in the aSD group (Fig. 3i, arrows). c-Fos expression was not detected in control input neurons (Fig. 3i, control). We next used a chronic social defeat (CSD) model to examine changes in the activity of the vmPFC-PVN^CRH^ circuit and associated behavioral alterations (Fig. 3j). Mice with RV-labeled input neurons were subjected to 10 consecutive days of CD-1 aggression and then assessed for social interaction and depression-like behaviors. FosB immunofluorescence in the mPFC revealed that, compared with control mice, defeated mice exhibited reduced social interaction (Fig. 3k, left) and significantly increased immobility in the forced swimming test (Fig. 3k, right). Similar to the response to acute defeat, FosB expression was significantly elevated in mPFC inputs after CSD (Fig. 3l). Data quantification confirmed a substantially greater proportion of FosB^+^ input neurons in the CSD group than in the control group (Fig. 3m).

Together, these results demonstrate that both acute and chronic SD stress activate the vmPFC-PVN^CRH^ circuit, which exhibits dynamic activity patterns in mice subjected to acute stress.

### vmPFC-PVN^CRH^ circuit mediates the bidirectional control of social avoidance behavior following aSD stress

**Fig. 4:**
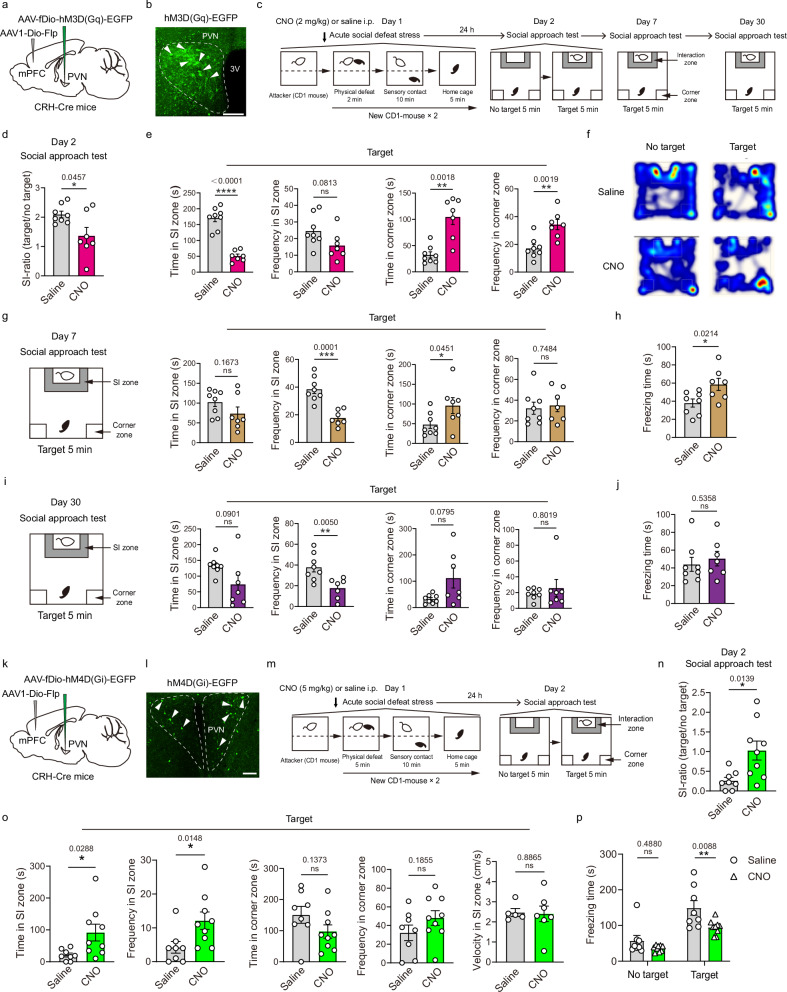
Chemogenetic manipulation of the vmPFC-PVN^CRH^ pathway modulates social stress-induced avoidance. **a** Viral strategy for chemogenetic activation of the vmPFC-PVN^CRH^ pathway. **b** Representative image of hM3D(Gq)-EGFP expression in PVN CRH neurons. Scale bar: 100 μm. **c** Experimental timeline: mice received saline or CNO (2 mg/kg) 30 min before aSD, followed by social approach tests on Days 2, 7, and 30. **d**, **e** Comparison of social avoidance behaviors (Day 2) between the saline- and CNO-treated groups. **f** Representative locomotor tracks from the social approach test. **g** Comparison of social avoidance behaviors (Day 7). Left: schematic of the test. **h** Comparison of freezing times (Day 7). **i** Comparison of social avoidance behaviors (Day 30). Left, schematic of the test. **j** Comparison of freezing times (Day 30). *n* = 8 mice (Saline group); *n* = 7 mice (CNO group). **k** Viral strategy for chemogenetic inhibition of the pathway. **l** Representative image of hM4D(Gi)-EGFP expression in PVN^CRH^ neurons. Scale bar: 100 μm. **m** Experimental timeline for pathway inhibition prior to aSD and social testing. **n**, **o** Social avoidance behaviors following pathway inhibition. **p** Freezing time following pathway inhibition. *n* = 7 mice per group. ^∗^*P* < 0.05, ^∗∗^*P* < 0.01, ^∗∗∗^*P* < 0.001, ^∗∗∗∗^*P* < 0.0001. The data are presented as the mean ± SEM. See Supplementary Table S1 for statistical details.

To establish a causal relationship between vmPFC-PVN^CRH^ circuit activity and psychosocial stress-related behaviors, we performed social interaction assays while the targeted circuit was manipulated. First, to specifically activate the vmPFC-PVN^CRH^ circuit, we used a viral strategy to express Gq-coupled receptors in CRH neurons within the PVN that receive predominant innervation from the vmPFC. We injected AAV1-Dio-Flp into the vmPFC and AAV-fDio-hM3D(Gq)-EGFP into the PVN of CRH-Cre mice (Fig. 4a), enabling chemogenetic activation of these specific CRH neurons (Fig. 4b, arrowheads). Neurons were activated by intraperitoneal injection of CNO (2 mg/kg) 30 min prior to behavioral testing. Initial open field (OF) and elevated plus-maze (EPM) tests revealed that circuit activation did not significantly affect general locomotion or anxiety-like behaviors (Supplementary Fig. S6a, b).

In subsequent experiments, a separate cohort of mice received CNO 30 min prior to aSD stress, which was followed by social approach testing at 24 h, 7 days, and 30 days post-stress (Fig. 4c). Control mice underwent identical procedures but received saline instead of CNO. On Day 2, the CNO-treated mice had a lower social interaction (SI) ratio (Fig. 4d). They also spent less time in the SI zone and entered it less frequently, but spent more time in the corner zone and entered it more frequently compared with controls when an aggressor was present (Fig. 4e). Representative heatmaps illustrated distinct behavioral trajectories in the CNO group (Fig. 4f). On Day 7 (Fig. 4g, h) and Day 30 (Fig. 4i, j) poststress, the CNO-treated mice continued to display increased social avoidance, characterized by reduced time spent and fewer entries in the SI zone, as well as more time in the corner zone. Furthermore, on Day 7, compared with control mice, mice in the CNO group exhibited significantly longer freezing times when confronted with a CD-1 threat (target) (Fig. 4h), although this difference was no longer present by Day 30 (Fig. 4j). We also assessed the potential off-target effects of CNO by comparing social interaction behaviors between EGFP-expressing mice treated with saline and those treated with CNO in the social approach test. No significant differences were found in the SI ratio, time spent, or entry frequency in the interaction/corner zones, or freezing time (Supplementary Fig. S6c).

These results indicate that premature overactivation of this circuit leads to prolonged social avoidance and negative emotional states, as reflected by increased freezing during the posttrauma period.

To assess the necessity of the vmPFC-PVN^CRH^ circuit in social avoidance, we inhibited the circuit by expressing Gi-coupled receptors in vmPFC-innervated CRH neurons within the PVN via the Gi-GPCR pathway. AAV1-Dio-Flp and AAV-fDio-hM4D(Gi)-EGFP were injected into the vmPFC and PVN of CRH-Cre mice, respectively (Fig. 4k), enabling selective chemogenetic inhibition of these neurons (Fig. 4l, arrowheads). Mice received CNO (5 mg/kg, i.p.) 30 min before aSD stress (5 min per round), and social approach was tested 24 h later (Fig. 4m). Compared with saline-treated control mice, CNO-treated mice presented a significantly greater SI ratio upon CD-1 exposure (Fig. 4n). They also spent more time in the SI zone and entered it more frequently, although no significant differences were observed in either the time spent or the frequency of entries into the corner zone. In addition, the average nose point velocity within the contact area was comparable to that of the controls (Fig. 4o). Furthermore, when a CD-1 threat was present, compared with control mice, CNO-treated mice exhibited significantly less freezing (Fig. 4p, target), whereas no difference was observed in the absence of the threat (Fig. 4p, no target). Like in the activation experiments, circuit inhibition did not significantly affect locomotion or anxiety-like behaviors in the OF or EPM tests (Supplementary Fig. S6d, e).

These findings demonstrate that preemptive inhibition of this circuit before threat exposure effectively reverses aSD-induced avoidance behaviors.

### Activation of the vmPFC-PVN^CRH^ circuit potentiates fear learning and impairs fear extinction

**Fig. 5:**
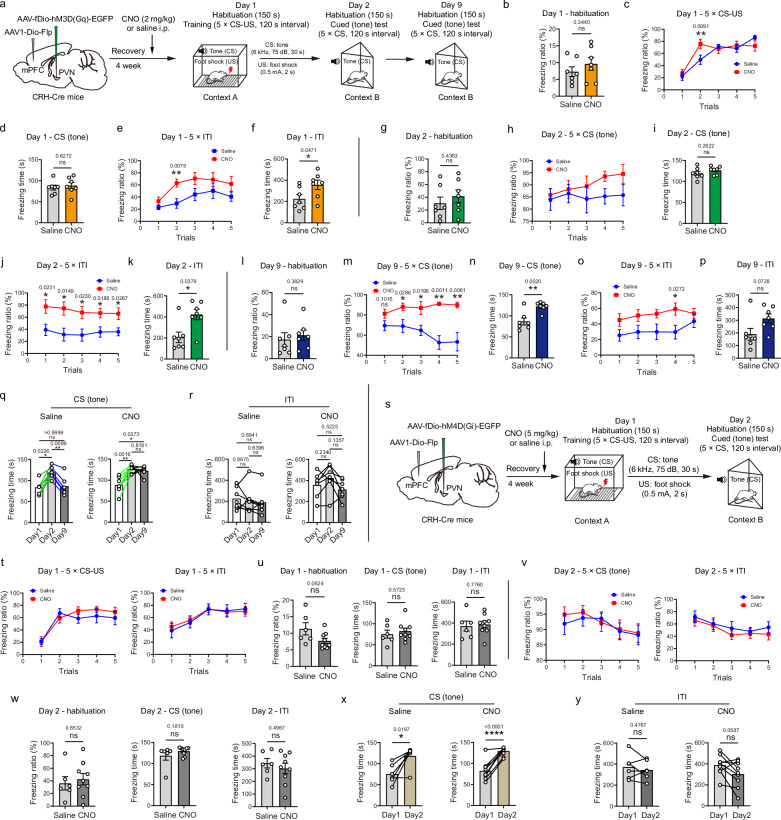
Chemogenetic activation of the vmPFC-PVN^CRH^ pathway enhances fear memory. **a** Viral strategy and experimental timeline for chemogenetic activation in the fear conditioning paradigm. **b**–**f** Freezing behavior in context A during conditioning (Day 1) in saline- vs CNO-treated mice. **g**–**k** Freezing behavior in context B during recall (Day 2) following chemogenetic activation. **l**–**p** Freezing behavior in context B during recall (Day 9) after chemogenetic activation. **q**, **r** Comparison of freezing across Days 1, 2 and 9 in the saline and CNO groups. **s** Viral strategy and timeline for chemogenetic inhibition in fear conditioning. **t**, **u** Effect of chemogenetic inhibition on freezing in context A during conditioning (Day 1). **v**, **w** Effect of chemogenetic inhibition on freezing in context B during recall (Day 2). **x**, **y** Comparison of freezing on Day 1 vs Day 2 in the saline and CNO groups. *n* = 6 mice (saline group); *n* = 9 mice (CNO group). ^∗^*P* < 0.05, ^∗∗^*P* < 0.01, ^∗∗∗∗^*P* < 0.0001; ns, not significant. The data are presented as the mean ± SEM. See Supplementary Table S1 for statistical details.

Fear conditioning is a widely studied model for re-experienced symptoms of PTSD^4,7^. We therefore investigated the impact of chemogenetic manipulation of the vmPFC-PVN^CRH^ circuit on fear behavior in mice. On the basis of a modified classical fear conditioning paradigm^8,10,44^, we first examined the effects of activating the vmPFC-PVN^CRH^ circuit on fear acquisition and extinction in mice subjected to tone-shock pairing (Fig. 5a).

On the first day of fear acquisition, the mice were intraperitoneally injected with CNO or saline before testing. No significant difference was observed in the freezing rate during habituation to the environment (context A) between the CNO and saline groups (Fig. 5b). However, during the conditioned stimulus (CS) stage, compared with saline-treated mice, CNO-treated mice experienced an earlier onset (trial 2) of increased freezing duration (Fig. 5c). The total freezing time during the CS (tone) stage did not differ significantly between the two groups (Fig. 5d). Notably, compared with saline-treated mice, CNO-treated mice exhibited higher freezing ratios within each intertrial interval (ITI) (Fig. 5e), and their total freezing time during these ITIs was significantly longer (Fig. 5f). This effect was not blocked by the corticosterone receptor antagonist mifepristone (Supplementary Fig. S7a).

On the second day after training, both groups showed similar habituation freezing ratios in a novel context B (Fig. 5g), and no significant differences were observed in freezing behavior during the CS (tone) phase (Fig. 5h, i). However, CNO-treated mice continued to display increased freezing durations during the ITIs on Day 2 (Fig. 5j, k), which was attenuated in the mifepristone + CNO group compared with that in the CNO-only group (Supplementary Fig. S7b).

To assess whether the vmPFC-PVN^CRH^ circuit is involved in the extinction of conditioned fear, both groups underwent an extinction retrieval test in the same environment on Day 9 (Fig. 5a). No significant difference in freezing was detected during the pre-CS exploration phase (Fig. 5l). Across five consecutive 30-s tone trials, compared with saline-treated control mice, CNO-treated mice maintained persistently higher freezing ratios (Fig. 5m). The total freezing time during the CS (tone) stage was also significantly greater in the CNO group (Fig. 5n), and these mice continued to show increased freezing during the ITIs on Day 9 (Fig. 5o, p). Compared with their own baselines, both groups showed a significant increase in total freezing time during the CS (tone) extinction phase on Day 2 relative to that on Day 1 (Fig. 5q). By Day 9, the total freezing time of the saline group was significantly shorter than that on Day 2, but the levels did not differ significantly from those on Day 1 (Fig. 5q, left). In contrast, compared with the control group, the CNO group maintained a significantly greater total freezing time than that on Day 1 (Fig. 5q, right). While the freezing duration in the saline group progressively decreased during the ITIs, that in the CNO group remained high throughout the extinction phase (Fig. 5r). The fear-promoting effect of circuit activation was likewise attenuated by mifepristone pretreatment on Day 9 (Supplementary Fig. S7c, d).

To evaluate the similarity between the fear phenotype induced by circuit activation and PTSD-like behavior, we used a modified single prolonged stress and electric foot shock (SPS&S) mouse model^9^ and compared fear-related behaviors among four groups: control (Saline), SPS&S (Saline SPS&S), circuit activation (CNO), and circuit activation plus SPS&S (CNO SPS&S). During the ITIs on Day 1 (fear learning) and Day 2 (fear expression), the freezing levels of the CNO group were comparable to those of the SPS&S group and significantly greater than those of the control (Saline) group, whereas the freezing level of the CNO SPS&S group was the greatest (Supplementary Fig. S7e). On Day 9, both the CNO and CNO SPS&S groups maintained total freezing times similar to the elevated levels observed in the SPS&S group during both tone presentation and the ITIs (Supplementary Fig. S7f).

These results indicate that the preactivation of the circuit not only facilitates fear learning but also impairs fear extinction, ultimately leading to PTSD-like behavior.

To investigate the necessity of the circuit in fear learning and extinction, we performed circuit inhibition experiments. In CS-US coupled with fear conditioning (Fig. 5s), no significant differences were observed between the groups on Day 1 in terms of the freezing ratio during habituation, CS (tone) presentation, or ITIs, or in total freezing time during the CS (tone) or ITIs (Fig. 5t, u). During extinction on Day 2, both groups showed comparable fear behavior at each stage (Fig. 5v, w). Within-group comparisons revealed that all mice exhibited an increase in total freezing time during the CS stage on Day 2 relative to that on Day 1 (Fig. 5x), whereas both groups showed reduced freezing durations during the ITIs on Day 2 compared with that on Day 1 (Fig. 5y). We also evaluated the potential off-target effects of CNO by comparing fear-related behaviors in EGFP-expressing mice treated with saline or CNO, and no significant differences in freezing behavior were observed between the two groups (Supplementary Fig. S8).

These results indicate that preemptive inhibition of this circuit prior to threat exposure does not affect conditioned fear responses.

## Discussion

Our study revealed a top-down monosynaptic glutamatergic pathway from the vmPFC to PVN^CRH^ neurons, which directly modulates psychological stress susceptibility and elicits PTSD-like phenotypes in mice. Our findings redefine one aspect of the role of the vmPFC in stress regulation and propose a neural substrate that could underlie differential vulnerability to stress-related disorders.

### Anatomical and functional specificity of the vmPFC-PVN pathway

The mPFC has long been proposed to exert inhibitory effects on endocrine responses to psychological stress^45–48^. Importantly, traditional anatomical studies have failed to demonstrate direct projections from the mPFC to the PVN^49,50^. In this study, the identification of direct vmPFC^Glu^-PVN^CRH^ monosynaptic projections adds novel circuit evidence to the classical model of mPFC-mediated indirect inhibition of the HPA axis via relay nuclei. Our viral tracing and single-neuron reconstruction data reveal that vmPFC layer 5/6 pyramidal neurons form axonal boutons directly apposing PVN^CRH^ somata, bypassing intermediate structures. This finding aligns with recent works^35–37^, in which PVN^CRH^ neurons were shown to integrate prefrontal inputs to coordinate stress-related behaviors. However, our functional data extend these findings by demonstrating that the activation of vmPFC terminals drives sequential calcium dynamics in PVN^CRH^ neurons, with “pacemaker” cells initiating population-level excitation. This hierarchical activation suggests that a subset of CRH neurons in the PVN may act as hubs to amplify stress signals, similar to the lateral habenula “starter cells” reported in depression models^51^.

Notably, our findings show that activating vmPFC-PVN projections increases the activity of CRH neurons, which aligns with recent reports. For instance, projections from mPFC pituitary adenylate cyclase-activating polypeptide (PACAP) neurons to the PVN are required for both CRH mRNA synthesis and sustained corticosterone elevation during acute restraint stress^36^. Consistent with this, optogenetic stimulation of mPFC neurons has been shown to induce c-Fos expression in the PVN^37^. In addition, we observed that chemogenetic activation of this pathway prior to FS stress elevates corticosterone levels compared to FS alone without circuit activation, which is in agreement with the findings of earlier studies^36–38^. However, chemogenetic activation of the vmPFC-PVN circuit under stress-free conditions did not significantly increase corticosterone levels. We propose that although this manipulation increases CRH neuronal activity, it may be insufficient to trigger substantial CRH peptide release under basal conditions, instead potentially lowering the threshold for peptide release upon subsequent stress exposure. Thus, the primary effects of the pathway may arise from local PVN circuit dynamics, and this pathway retains the capacity to amplify neuroendocrine stress responses when activated in concert with a salient threat.

### Circuit hyperactivity mimics PTSD-like phenotypes

The input neurons we identified are predominantly located within the vmPFC, a brain region that provides inhibitory control over threat-related memories and behaviors in the context of PTSD^18,52–54^. A striking finding of our study is that activation of the vmPFC-PVN^CRH^ circuit prior to stress exposure predisposed mice to prolonged social avoidance and fear memory persistence. This finding aligns with clinical observations linking prefrontal hyperactivity to PTSD vulnerability^4^. For instance, PTSD patients exhibit heightened mPFC activation during trauma recall^24^, whereas rodent studies have shown that mPFC lesions reduce stress-induced freezing^46^. Interestingly, our results from the aSD experiments suggest that in response to a sudden threat (first attack on Day 1), the vmPFC input neurons showed low activity; as the stress persisted, the increased activity of vmPFC input neurons occurred in the subsequent attack moment, upon repeat interactions, and when the mice remained alone in the corner area on Day 2. We posit that in response to a threat, the elevated activity of PVN^CRH^ neurons constitutes an instinctive mechanism for processing bottom-up stress-induced information. During this phase, the mPFC inputs exhibit reduced activity, characterized by disconnected PVN^CRH^ neurons and reduced energy utilization. Subsequently, the increased activity of mPFC inputs reflects the perception and deeper processing of sustained stress-related information. In addition, the time-lagged cross-correlation suggested that vmPFC input activity increased prior to PVN neuron activity during social contact on Day 2. Thus, the dynamic interplay between the activities of mPFC inputs and PVN^CRH^ neurons during the threat stress response process illustrates a top-down cognitive amplification mechanism, wherein higher-order cortical circuits subjectively enhance the salience encoding of prior threatening experiences, akin to the re-experiencing symptoms observed in PTSD^4^.

Our data further suggest that experimentally induced hyperactivity in this pathway prior to trauma exposure can prime maladaptive stress responses. This is supported by the lack of baseline anxiety changes in OF/EPM tests following circuit manipulation, indicating specificity to threat processing rather than general emotionality. In the manipulation experiments, the freezing behavior characterized by nasal movement velocity dynamics (quantified by sniffing speed) in the SD tests and freezing time in the ITIs were included in the analysis, which could provide critical mechanistic insights into emotional discrimination during the social interaction or fear process. In conditioned fear testing, preactivation of this circuit not only facilitates fear acquisition during conditioning but also impairs extinction learning. These findings align with the behavioral profile observed following trauma exposure in both humans with PTSD and rodent models^55^, which is further evidenced by the comparison between our circuit‑based manipulation and the SPS&S model. Intriguingly, during our fear extinction test, mice exhibited increased freezing behavior specifically during the ITIs of auditory cue presentation. We propose that this temporal window represents a critical period for fear memory reconsolidation within threat-processing networks. Corticosterone antagonism experiments revealed that while activation of the circuit (CNO group) robustly increased freezing during ITIs and impaired fear extinction, these effects were attenuated in the group that received both CNO and mifepristone. These findings indicate that the fear-promoting actions of the vmPFC-PVN^CRH^ circuit are, at least in part, dependent on glucocorticoid receptor signaling. This finding aligns with the established role of corticosterone in fear memory consolidation and suggests that the circuit may promote fear processes by facilitating a corticosterone-dependent mechanism, possibly owing to its capacity to increase corticosterone levels under stress. Furthermore, the persistence of avoidance behaviors for 30 days post-stress and failed fear extinction parallels the chronicity of PTSD symptoms and suggests circuit-driven synaptic plasticity. Thus, an artificially hyperactive vmPFC-PVN^CRH^ circuit response to threats can model a state of vulnerability. Future studies are needed to determine whether intrinsic, preexisting hyperactivity in this circuit serves as a natural biomarker for susceptibility in unmanipulated individuals.

### Divergent roles in social avoidance vs conditioned fear

The distinct differences observed in our manipulation experiments, where inhibition of the vmPFC^Glu^-PVN^CRH^ circuit reversed aSD-induced avoidance but spared both the acquisition and expression of conditioned fear, suggest that this functional separation arises from distinct cognitive and neural demands of the two behavioral paradigms. SD is not merely a physical stressor but also a potent psychosocial event involving the dynamic evaluation of social threat, submission, and hierarchical dynamics^41^, although the psychological mechanisms through which it elicits various physiological responses remain unclear^23^. Our data indicate that the vmPFC^Glu^-PVN^CRH^ pathway may be selectively recruited to encode the salience and motivational value of psychosocial threats, likely through direct excitation of PVN^CRH^ neurons, thereby promoting a state that facilitates avoidance and withdrawal from contexts perceived as socially dangerous — which is consistent with the social withdrawal symptoms observed in individuals with PTSD^6^. In support of this, our in vivo calcium recording data revealed that vmPFC input neurons are dynamically engaged during social investigation and retreat, which are moments rich in social decision-making. Conversely, circuit inhibition may attenuate the consolidation of social threat signaling, thereby restoring normal social interaction.

The formation, storage, and expression of auditory-cued fear are governed by a well-characterized amygdala–hippocampal–prefrontal circuit^56^. We propose that the excitatory and inhibitory outputs of this circuit differentially modulate PVN neuronal activity and HPA axis function, driving distinct corticosterone-dependent effects on conditioned fear behavior. This interpretation is supported by corticosterone measurements following circuit manipulation: circuit preactivation increased corticosterone levels under FS stress, whereas circuit inhibition failed to reverse this change. Further support comes from the behavioral effects of corticosterone receptor blockade during circuit activation, in which mifepristone restored impaired fear extinction without altering fear acquisition. Together, these results suggest that circuit inhibition does not abolish immediate HPA axis activation or corticosterone-mediated behavioral effects in the context of conditioned fear.

Future studies should examine how activity in this pathway differs in response to social versus physical threats and explore its functional interplay with canonical amygdala‑based fear circuits.

### Therapeutic implications and translational outlook

The discovery of a discrete, top-down vmPFC^Glu^-PVN^CRH^ circuit refines our understanding of prefrontal control over stress responses. While clinical models emphasize generalized mPFC inhibition of the HPA axis, this direct excitatory pathway provides a specific substrate for heightened stress reactivity, potentially contributing to phenotypes such as hypocortisolism in PTSD^42,57^. This circuit-level insight addresses the limitations of human neuroimaging and neuroendocrine studies. Given that it is functionally specialized for psychosocial threat salience and persistent avoidance (distinct from cued fear memory), this pathway represents a precise target for intervention. Future neuromodulation or therapies restoring prefrontal–hypothalamic balance may alleviate maladaptive avoidance while preserving adaptive fear. The “pacemaker” properties of specific PVN^CRH^ neurons further suggest cell type-specific strategies. Ultimately, identifying noninvasive biomarkers of the activity of this circuit could bridge these mechanistic findings to human vulnerability stratification and treatment monitoring.

### Limitations and future directions

Our investigation was conducted exclusively in male mice. Given the well-established sex differences in the prevalence, symptomatology, and neurobiology of PTSD in humans^55^, this represents a constraint on the generalizability of our findings. Sex differences in prefrontal–amygdala circuit activity may shape adaptive fear responses across sexes. Trauma could further polarize this function, contributing to sex-specific PTSD features. While rodent studies have shown that there are sex differences in fear conditioning, evidence for differences in fear learning and extinction remains inconsistent, likely because of unaccounted gonadal hormone status^58^. Future studies are essential to determine whether the vmPFC^Glu^-PVN^CRH^ circuit operates similarly in females and how ovarian hormone cycles might modulate its function and behavioral output. Investigating this pathway in both sexes will be crucial for understanding the neural basis of sex-specific vulnerability and for developing equitable therapeutic strategies.

In conclusion, we identified a unique top-down neural circuit whose activity regulates the development of PTSD-like behaviors in mice. This circuit offers distinctive insights into the pathogenesis of neuropsychiatric disorders and might serve as a novel target for the treatment of related diseases.

## Materials and methods

### Animals

Male C57BL/6J (stock number 000664, The Jackson Laboratory), CRH-IRES-Cre (stock number 012704, The Jackson Laboratory), Rosa26-pCAG-LoxP-stop-LoxP-tdTomato-P2A-G-IRES-TVA (a gift from Drs. Lingqiang Zhu and Youming Lu) and Ai14 (a gift from Dr. Minmin Luo) mice, aged 6–12 weeks, were used in this study. The CRH-IRES-Cre driver line was fully backcrossed to the C57BL/6J background. The mice in each cohort were matched for age (± 1 week) and body weight (± 2 g) across all the control and treatment groups to ensure comparability. The mice were individually housed under a 12-h light/dark cycle with ad libitum access to food and water (ambient temperature: 22 °C–24 °C; humidity: 50%–60%). All behavioral experiments were conducted during the light phase. Prior to experimentation, the animals were habituated to handling through daily 5-min manual restraint sessions for 3 consecutive days. For the SD stress test, retired male CD-1 (also known as ICR) breeder mice (8 months of age; Vital River, China) were used. The mice were randomly assigned to experimental groups. For behavioral, histological, and calcium imaging experiments, investigators were blinded to group assignment (viral treatment or drug administration) during data acquisition and analysis. All animal procedures were approved by the Anhui Medical University Institutional Animal Care and Use Committee (IACUC) and conducted in accordance with institutional guidelines (LLSC20241768).

### Viral vectors

For the neuronal tracing and labeling experiments, we used RV-EnvA-ΔG-EGFP/dsRed, AAV9-EF1α-Dio-H2B-BFP-T2A-TVA, AAV9-EF1α-Dio-RVG, AAV1-EF1α-Cre-EGFP, and AAV-NSSP-EGFP-1E5(Flp). For in vitro optogenetic and Cre-dependent GCaMP6m expression, we used AAV9-hSyn-CaMK2-hChR2(H134R)-EYFP, AAV9-hSyn-CaMK2-ChrimsonR-mCherry, and AAV9-CAG-Dio-GCaMP6m. For chemogenetics, we used AAV1-hSyn-Dio-FLP, AAV8-hSyn-fDio-hM3D(Gq)-EGFP and AAV8-hSyn-fDio-hM4D(Gi)-EGFP and AAV8-hSyn-fDio-EGFP. To induce retrograde Cre-dependent GCaMP6s expression, we used AAV9-EF1α-Dio-TVA-BFP, AAV9-EF1a-Dio-RVG and RV-CVS-EnvA-N2C(ΔG)-GCaMP6s. All viruses were purchased from Brain VTA, Wuhan, or Brain Case, Shenzhen.

### Surgery

The mice were deeply anesthetized using 5% isoflurane in oxygen-enriched air (O_2_ Concentrator, RWD, Shenzhen, China) and then fixed in a stereotaxic frame (RWD Life Science, Shenzhen, China). Anesthetized mice were maintained on 2%–2.5% isoflurane, and the core body temperature was maintained at 36 °C using a feedback-controlled temperature controller (ATC2000; RWD Life Science, Shenzhen, China). Eye ointment was applied to prevent dryness. The head was shaved, and the skin was sterilized using iodine solution. Afterward, a midline incision was made with a scalpel to expose the skull.

### Injections

For viral vectors, approximately 0.02–0.3 μL per hemisphere was injected using pulled glass pipettes (tip diameter 10–20 μm, PC-100 puller) connected to a pressure ejector (Micro 4, WPI). The PVN coordinates used were –0.82 mm posterior to the bregma, ± 0.25 mm lateral to the midline, and 4.4–4.6 mm below the dura. The mPFC coordinates used were 1.7–1.8 mm anterior to the bregma, ± 0.3–0.4 mm lateral to the midline, and 1.0–2.0 mm below the dura (1.0–1.4 mm for the dmPFC, 1.5–2.0 mm for the vmPFC). For CRH staining, 0.5 μl of colchicine (0.2 mg/kg) was injected into the lateral ventricle (LV). The LV coordinates used were –0.5 mm posterior to the bregma, ±1.0 mm lateral to the midline, and 1.7–1.8 mm below the dura.

### Fiber implantation for recording calcium activity

Animals were implanted with LC optic fiber stubs (fiber: 0.37 NA, 200 μm diameter, 4–6 mm length; Thinker Tech, Nanjing) 2–3 weeks after viral vector injection. Optic fiber tips were lowered to 100–200 μm above the injection site of the PVN or mPFC. The implants were fixed to the skull using screws and dental cement. Experiments were conducted 4 weeks after viral infection.

### Histology

The mice were deeply anesthetized and transcardially perfused with PBS, followed by 4% paraformaldehyde (PFA) dissolved in 0.1 M PB (pH 7.4). The brains were removed and postfixed overnight at 4 °C in 0.1 M PB containing 4% PFA. For whole-brain imaging, brain samples were processed either by impregnation with glycol methacrylate (GMA; Ted Pella Inc.) and vacuum oven embedding for fMOST^59,60^ or by transparent processing using clearing solution for VISoR^61,62^. To visualize fluorescently labeled neurons and immunofluorescence, the fixed brains were embedded in agarose, and consecutive 50- or 100-μm-thick coronal sections were collected using a vibrating microtome (Leica VT1200S, Germany). The sections were subsequently washed in PBS and permeabilized with 0.3% Triton X-100 for 30 min, followed by blocking with 5% normal donkey serum at room temperature for 1 h. The sections were then incubated with the primary antibody in PBS containing 0.3% Triton X-100 overnight or for 36 h (CRH immunostaining, using an anti-CRF antibody) at 4 °C. The following primary antibodies were used: rabbit anti-CRF (1:2000; Bachem, T4037), rabbit anti-c-Fos (1:1000; Cell Signaling Technology, #2250), and mouse anti-FosB (1:100; Santa Cruz Biotechnology, sc-398595). After being washed in PBS, the sections were incubated with secondary antibodies (DyLight 405- or Alexa Fluor 647-conjugated donkey anti-rabbit or Alexa Fluor 488-conjugated donkey anti-mouse, Jackson ImmunoResearch, 711-475-152, 711-605-152, or 715-545-150) diluted 1:200 in 0.1% Triton X-100 in PBS at room temperature for 2 h. After being washed in PBS, the sections were mounted on slides with 80% glycerol solution and stored at –20 °C. All the sections were photographed using an LSM 880 confocal microscope (Zeiss, Germany) equipped with a 10× or 20× objective. Confocal images were acquired, and cell counting was performed using ImageJ software (NIH) or Imaris 9.0 software (Bitplane, Switzerland). Anatomical regions were identified according to the Brain Atlas of Paxinos & Franklin (2001) and the Allen Institute Mouse Brain Atlas.

### Blood sampling and corticosterone level measurements

For blood collection, the distal tip of the mouse tail was excised, and blood was collected incrementally (2–3 samples) until a volume of 10 μL was obtained^63^. Following serum separation (centrifugation at 3000 rpm for 15 min at 4 °C), the samples were stored at –80 °C until analysis. Cage-based randomization was applied during sampling to prevent temporal biases in corticosterone measurements. The mice remained in their home cages in a low-stress environment (noise-minimized room) throughout the experiment. Serum corticosterone was assayed in 5 μL aliquots using an ELISA kit (EIACORT, Thermo Fisher) according to the manufacturer’s instructions.

### Whole-brain imaging and neuronal reconstruction

Brain samples for neuronal tracing in mice were imaged using the fMOST^59^ or VISoR^61^ technique. Briefly, the fluorescent protein-labeled neuron datasets of the mouse brain were acquired from 1 µm thick serial brain sections at a voxel resolution of 0.32 μm × 0.32 μm × 1 μm (fMOST) or 4550 300-µm sections at a voxel resolution of 0.5 μm × 0.5 μm × 2.5 μm. After image processing, the reconstruction of the input or output neurons was completed interactively with Amira visualization and data analysis software (Version 5.4.3; Thermo Fisher Scientific, USA) or Imaris software (Version 9.0.1; Bitplane, Switzerland). The datasets were converted into a large disk data object, and the fibers in each subvolume were traced interactively. To characterize the input neurons derived from the mPFC, the 2D coordinates of the pyramidal neuron somata were determined and subsequently mapped onto the 3D subvolumes. The complete dendritic and axonal arborizations of these neurons were then reconstructed. For anterogradely sparsely labeled neurons, fiber terminals within the PVN that made synaptic contact with CRH^+^ somata were first identified and then retrogradely traced back to their mPFC somata for complete axonal projection pathway reconstruction. The axon terminals marked by white arrowheads in Fig. 1q (from the VISoR datasets) and the synaptic boutons shown apposing CRH neuron somata in the inset of Fig. 1p (from post-confocal microscopy images) are derived from the same PVN sample. The reconstructed neurons were examined sequentially by 3 people.

### In vitro electrophysiology and calcium imaging

The mice were anesthetized with sodium pentobarbital (80 mg/kg, i.p.) and decapitated. The brains were transferred to an ice-cold saturated oxygen cutting recovery solution containing 93 mM NMDG, 2.5 mM KCl, 1.2 mM NaH_2_PO_4_, 30 mM NaHCO_3_, 20 mM HEPES, 25 mM glucose, 5 mM sodium ascorbate, 2 mM thiourea, 3 mM sodium pyruvate, 10 mM MgSO_4_, and 0.5 mM CaCl_2_ (pH: 7.3–7.4; osmolarity: 300–305 mOsm/kg). Coronal slices (300 μm in thickness) of the PVN were prepared using a microtome, and all of the slices were transferred to cutting recovery solution. Slices were incubated at 32 °C for 10–15 min and then transferred to modified HEPES recovery solution containing 92 mM NaCl, 2.5 mM KCl, 1.2 mM NaH_2_PO_4_, 30 mM NaHCO_3_, 20 mM HEPES, 25 mM glucose, 5 mM sodium ascorbate, 2 mM thiourea, 3 mM sodium pyruvate, 2 mM MgSO_4_, and 2 mM CaCl_2_ (pH: 7.3–7.4; osmolarity: 300–305 mOsm/kg) at 28 °C for at least 60 min. Slices were then transferred to a recording chamber at room temperature (20 °C–24 °C) and constantly perfused with standard ACSF containing 127 mM NaCl, 25 mM NaHCO_3_, 25 mM D-glucose, 2.5 mM KCl, 1.25 mM NaH_2_PO_4_, 2 mM CaCl_2_, and 1 mM MgCl_2_ (pH: 7.4; osmolarity: 300–305 mOsm/kg). For patch clamp recording, target PVN^CRH^ neurons were identified by fluorescence. Glass electrodes (5–7 MΩ) were used for recording. The signals were amplified using a MultiClamp 700B amplifier (Molecular Devices, USA). Data were filtered at 2 kHz and digitized at 10 kHz (Digidata 1440, Molecular Devices). For optogenetic stimulation of the brain slices, an optical fiber (200 μm inner core diameter; Thinker Tech, Nanjing) coupled to a 473 nm diode-pumped solid-state laser (Thinker Tech, Nanjing) was submerged in ACSF and placed above the recording site. An illumination intensity of 10–15 mW/mm^2^ was used for 473 nm blue light irradiation. To validate the efficacy of opsin, td-Tomato^+^ CRH-expressing cells were held at –60 mV in voltage-clamp mode or at 0 pA in current-clamp mode. A train of 473 nm light pulses (5 ms width; 20 Hz) lasting for 400 ms or 1000 ms was used. The internal solution consisted of the following: 130 mM potassium gluconate, 5 mM KCl, 10 mM HEPES, 2.5 mM MgCl_2_, 4 mM Na_2_ATP, 0.4 mM Na_3_GTP, 10 mM sodium phosphocreatine and 0.6 mM EGTA (pH: 7.2; osmolarity: 280–290 mOsm/kg). To record the EPSCs, a high Cl^-^ intracellular solution containing 120 mM KCl, 30 mM NaCl, 5 mM EGTA, 10 mM HEPES, 1 mM MgCl_2_, 0.5 mM CaCl_2_ and 2 mM Mg-ATP (pH: 7.2; osmolarity: 280–290 mOsm/kg) was used. Neurons were held at -60 mV by using a voltage clamp. For calcium imaging and optogenetic stimulation, PVN^CRH^ neurons were identified by GCaMP fluorescence, and an optical fiber coupled to a 630 nm diode-pumped solid-state laser was submerged in ACSF and placed above the recording site. A train of light pulses (35 ms in width; 20 Hz) lasting for 5–10 s was used. For chemogenetic stimulation, CNO (5 μM in ACSF) was perfused into the brain slice chamber via a peristaltic pump at a flow rate of 10–20 rpm. The calcium activity of the neurons within the field of view was dynamically captured in real time using a camera (2.5–10 fps), both before and after illumination or CNO administration.

### Fiber photometry recording

To record calcium fluorescence signals emitted by GCaMP, a fiber photometry system (ThinkerTech, Nanjing) was employed^51,64^. Briefly, a laser beam generated by a 470 nm laser was reflected by a dichroic mirror at low power (20–40 μW) at the tip of the optical fiber to minimize bleaching and was maintained constant during each recording session. The fluorescence signals were bandpass filtered, converted to voltage signals, digitized at 100 Hz, and then recorded using a custom-written script in LabView. The animals that underwent fiber photometry recording were placed in their home cages and subjected to SD stress tasks. For optogenetic activation, a 630 nm laser was delivered to the fiber optic cannula via an additional optical port for concurrent fiber photometry recording. Behavior-coupled fluorescence events were determined by peristimulus time histograms, which were calculated as the ΔF/F or *Z* score.

### Calcium imaging processing

In vitro Ca^2+^ activity images were first aligned along the x-y plane in ImageJ. Individual regions of interest (ROIs) were manually constructed, and the average fluorescence intensity was calculated by a time series analyzer. Afterward, the average fluorescence intensity (F) for each ROI was normalized to its initial baseline value (F0) by calculating ΔF/F = (F – F0)/F0 for each time point in MATLAB. The baseline fluorescence (F0) for each cell was defined as the mean fluorescence intensity during the 30-s period immediately preceding light stimulation while the slice was continuously perfused with standard ACSF. This prestimulus window was free from any experimental manipulation and represented a stable, resting state. Photobleaching was minimized by using low illumination intensity and short exposure times, and its effect was mathematically corrected for during the ΔF/F calculation. Potential motion artifacts were monitored and excluded (mechanical disturbance during perfusion or stimulation and recordings with drift during the baseline and stimulation period), and nonresponding cells within the same field of view were used as internal controls. For in vivo data, MATLAB was used for calcium fluorescence signal analyses, which included plotting representative data, generating average trace or heatmap graphs, and calculating the peak values of ΔF/F (%) (change in fluorescence (ΔF) relative to basal fluorescence (F0)), Z score (averaged GCaMP6 signal (F) over 3 s just before the response to give a mean and standard deviation, and the data were transformed into (F – mean)/standard deviation), the AUC of Ca^2+^ activity events, and the matched time points. For displaying the data in time‑series plots, Z score traces from different recording sites were aligned to a common baseline reference (zero line) to facilitate visual comparison of event‑locked deviations. Ca^2+^ activity was identified as an event when its peak was greater than 1.5 standard deviations of the baseline. Correlation coefficients between mPFC inputs and PVN neuron traces were calculated for 8–10 s event periods. Time-lagged cross-correlation consisted of a series of correlation calculations carried out with one trace shifted relative to another over an 8–10 s window. Correlation and time lag boxplots were plotted in MATLAB or GraphPad.

### Behavioral assays

The behavioral experiments were performed between 10:00 am and 7:00 pm. The mice were handled for at least 3 days before each behavioral procedure. For chemogenetic activation or inhibition experiments, saline or CNO (2 mg/kg for activation or 5 mg/kg for inhibition experiments, dissolved in saline) was administered intraperitoneally 30 min prior to each behavioral test. During the behavioral tests, all of the mice were housed individually. In all tests, the observers were blinded to the genotype. For the forced swimming tests, the results were calculated by researchers who were blinded to the experimental conditions. For other behavioral tests, the results were calculated by EthoVision XT 12 software (Noulds, Netherlands).

### OF test

In the OF test, a white wooden box (50 cm × 50 cm × 25 cm) with smooth interior walls was used. The center area of the OF was defined as a 25 cm × 25 cm zone centered in the arena. The mice were placed in one of four corners, facing the wall and permitted to explore the environment freely for 6 min. Locomotion traces were recorded with a video camera. The time and number of entries in the center area and total distance traveled were analyzed.

### EPM test

The EPM was composed of two opposite open arms (30 cm × 6 cm), two opposite closed arms (30 cm × 6 cm ×15 cm), and one central zone (6 cm × 6 cm). The plus-shaped apparatus was elevated 80 cm above the ground. The mice were placed on the central platform facing one of the open arms and allowed to explore the maze for 6 minutes. Locomotion traces were recorded with a video camera. The time spent in and the number of entries into the open arms were quantified and analyzed.

### Forced swimming test

The apparatus was a transparent cylinder (25 cm high, 25 cm diameter) filled with water to a depth of 18 cm and maintained at 24 °C–25 °C. The mice were placed in the container with their back to the wall and with their front paws touching the water. After each session, the mouse was dried with a towel and returned to its home cage. The water was replaced with clean water after each test. The total immobility time over the course of 5 min was recorded and analyzed. The mice were considered immobile when they did not make any active movements.

### SD stress

The mice were subjected to either an acute or a chronic social defeat (aSD or CSD) stress paradigm according to previously described methods^64–67^ with slight modifications. Retired male CD-1 (ICR) breeder mice were screened for aggressive behaviors, and only mice that reliably attacked within less than 30 s in 3 consecutive tests were used as aggressors. In the aSD paradigm, an experimental mouse was placed in the home cage of a CD-1 aggressor for 2 min (activation experiment) or 5 min (inhibition experiment). During the exposure, the experimental mouse was physically defeated by the aggressor. Afterward, the experimental mouse was separated from the aggressor behind a protective perforated plexiglass barrier to allow sensory contact. After 10 min of sensory contact, the experimental mouse was returned to its home cage for 5 min, followed by a second defeat by a new CD-1 aggressor. After the two rounds of defeat and sensory contact, the defeated mouse was returned to its home cage and subjected to a social approach test 24 h, 7 days, and 30 days later. For chemogenetic manipulation, CNO (2 mg/kg for activation or 5 mg/kg for inhibition) was delivered 30 min prior to the two bouts of defeat. The doses were chosen on the basis of established protocols from the literature demonstrating the efficacy and specificity for each receptor type at these doses in SD stress tests^67^.

In the CSD paradigm, the experimental mouse was introduced into the home cage of an unfamiliar CD-1 resident for 5 min and was physically defeated. Following the 5-min defeat episode, the experimental mouse was separated by a protective perforated plexiglass barrier. After 20 minutes of sensory contact, the experimental mouse was returned to its home cage overnight until the next defeat by a new CD-1 aggressor. This repeated stress continued for 10 days. Nonstressed control mice were housed individually throughout the experiment and were not exposed to CD-1 animals until the social approach test. The social approach test was performed 24 h after the final defeat and sensory contact.

### Social approach test

The test was performed in a white Plexiglass OF (42 cm × 42 cm × 42 cm), with one plastic mesh target box (10 cm × 6.5 cm × 42 cm) placed on one side of the OF. The interaction zone of the test arena encompassed a 15 cm × 25 cm rectangular area around the box. In the first 5-min session, the experimental mouse was allowed to freely explore the OF with no social target (CD-1 mouse) (no target) in the box. After a short 1-min rest in the home cage, the experimental mouse was returned to the OF to explore for another 5 min with an unfamiliar CD-1 mouse (target) present in the box. Using EthoVision XT 12 software, the distance moved, velocity, time spent in the social interaction zone and corner, number of entries in each session, and freezing time were measured. The social interaction ratio^67,68^ was calculated by dividing the time spent in the interaction zone in the presence of a social target by the time spent in the interaction zone in the absence of the social target.

### PTSD model establishment (SPS&S stress paradigm)

The PTSD model was established using a serial stress protocol^9^ comprising four distinct stressors: restraint, forced swimming, deep anesthesia, and unconditioned FS. The mice in the experimental group were first subjected to 4 h of restraint stress in their home cages using a rodent restraint device. This was followed by a 30-min recovery period in a large cage. The mice were subsequently forced to swim for 20 minutes in a water-filled plastic tub (25 °C water maintained at a 20 cm depth), after which they recovered in their home cages for another 30 minutes. The mice were then deeply anesthetized with ether until the loss of response to tail and toe pinch was observed. Thirty min later, they received an unconditioned FS (0.8 mA, 5 s) in a chamber equipped with an electrified grid floor. Control mice were placed in an identical chamber for 5 s without receiving a shock to control for environmental exposure. For chemogenetic activation, CNO (2 mg/kg) was delivered 30 minutes prior to SPS&S. All the mice were then returned to their home cages and left undisturbed.

### Fear conditioning test

The conditioned fear test for assessing PTSD behavior was conducted with reference to previous studies^9,44^ with slight modifications. For the acquisition trial, the mice were habituated for 150 s followed by a 30 s (6 kHz, 75 dB) tone. A 0.5 mA shock coterminated during the last 2 s in context A. The tone/shock was repeated five times with a 120 s ITI. For the extinction-cued trial (24 h or 9 days later), the mice were habituated for 150 s in a new context (context B), and then a tone was played five times with 30 s ITIs. For chemogenetic manipulation, CNO (2 mg/kg for activation or 5 mg/kg for inhibition) was delivered 30 minutes prior to the Day 1 acquisition test. The freezing ratio and freezing time during the tones and intervals were quantified. Each cohort was randomized, and the experimenters were blinded to the treatments. The data were analyzed using commercial software (AniLab, Ningbo).

### Statistical analysis

All the data were evaluated using GraphPad Prism 10.0 (GraphPad, USA). Normality was assessed by the Shapiro‒Wilk test. Homogeneity of variance was assessed by the F test or Brown–Forsythe test. Data that met these two conditions were analyzed using a two-tailed unpaired or paired *t*-test, one-way analysis of variance (ANOVA), and two-way ANOVA. Datasets that were not normally distributed were analyzed with a nonparametric test. For datasets involving multiple measurements from the same animal (e.g., multiple neurons recorded from one mouse or repeated behavioral trials), the data were analyzed using animal-level averages to ensure statistical independence unless otherwise specified in the figure legends. For calcium imaging experiments involving multiple cells per slice, representative cells or slice-level summaries are presented as indicated. All the statistical details are described in the Supplementary Information (Table S1). The data are presented as the mean ± SEM. *P* < 0.05 was considered to indicate statistical significance.

## Supplementary information

Supplementary Information

## Data Availability

The data that support the findings of this study are presented in the paper and/or the Supplementary information.
