# Supplementary material for: Top-down medial prefrontal cortex-to-hypothalamic paraventricular nucleus circuit regulates social avoidance and conditioned fear in male mice
Source: Cell Discov. 2026 Jun 9;12:42. doi: 10.1038/s41421-026-00893-6 (PMC13250170; doi:10.1038/s41421-026-00893-6)
Supplement: Supplementary file 1 — Supplementary Information [file 41421_2026_893_MOESM1_ESM.pdf]

Supplementary information for

A top-down medial prefrontal cortex to hypothalamic paraventricular nucleus circuit regulates social avoidance and conditioned fear in male mice

Yu Wang [1]*, Xiang-Yu Pan [1], Bo Wu [1], Dan-Yang Li [1], Shuo-Wen Wang [1], Xin-Ya Qin [1], Qing-Hong Shan [1], Peng Chen [1], Pu Hu [2], Hao Wang [3,4], Rong-Yu Liu [5], Hui Gong [6] & Jiang-Ning Zhou [1,7]*

*: Corresponding author. Email: wangyu10@ustc.edu.cn or jnzhou@ustc.edu.cn

Supplementary Figures

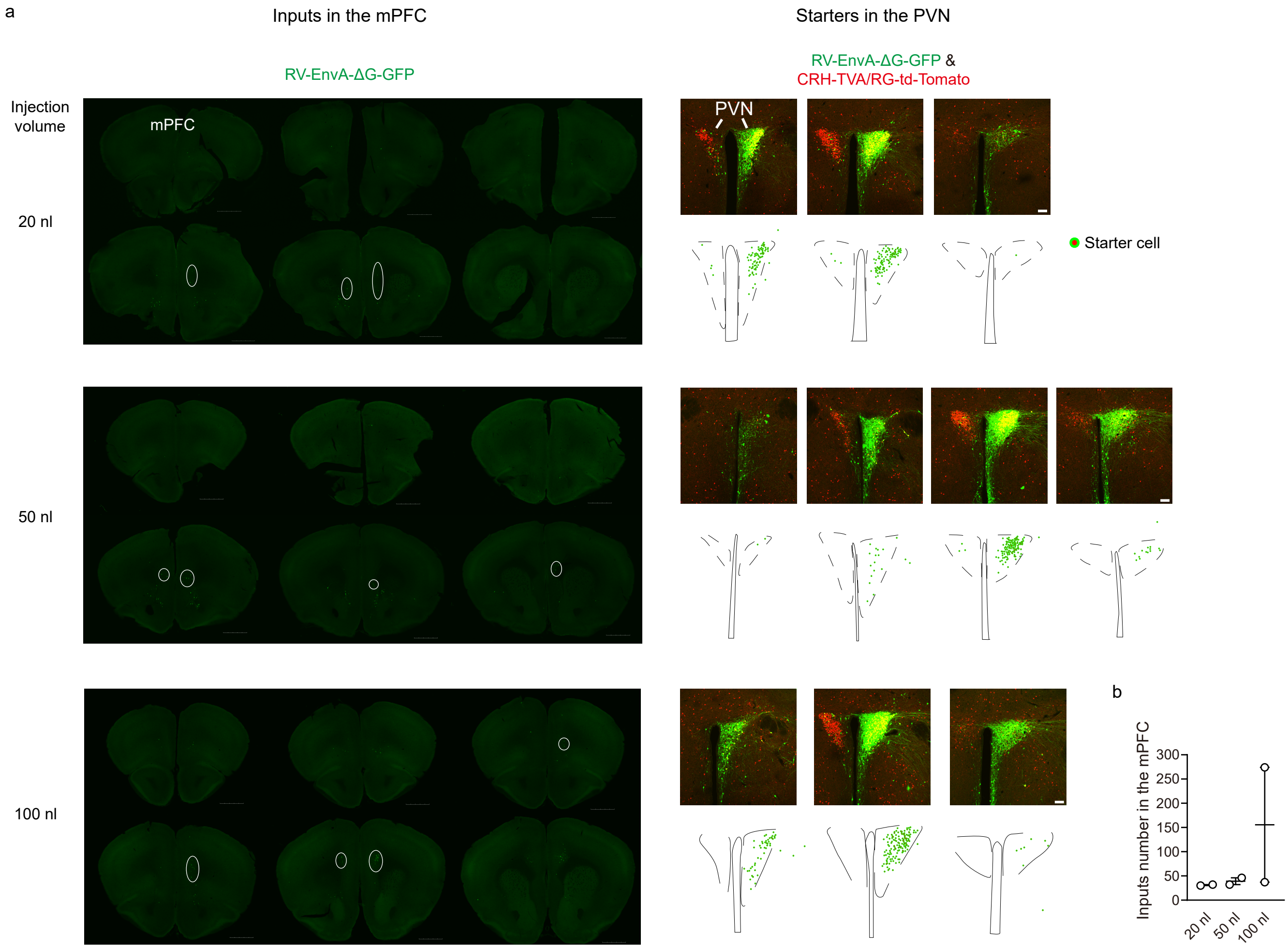

Supplementary Fig. S1. Inter-individual variations in the distribution of PVN starter neurons and mPFC inputs after injection of different viral doses using retrograde tracing.

(a) Representative images and schematic diagrams from consecutive sections show both mPFC inputs and PVN starter neurons in samples injected with 20 nL, 50 nL, or 100 nL of virus into the PVN. Circles in the mPFC images indicate the distribution of inputs within the PrL/IL subregion. **(b)** Statistical analysis of the number of mPFC inputs under different viral injection volumes into the PVN. Scale bars: 1 mm (mPFC images), 50 µm (PVN images).

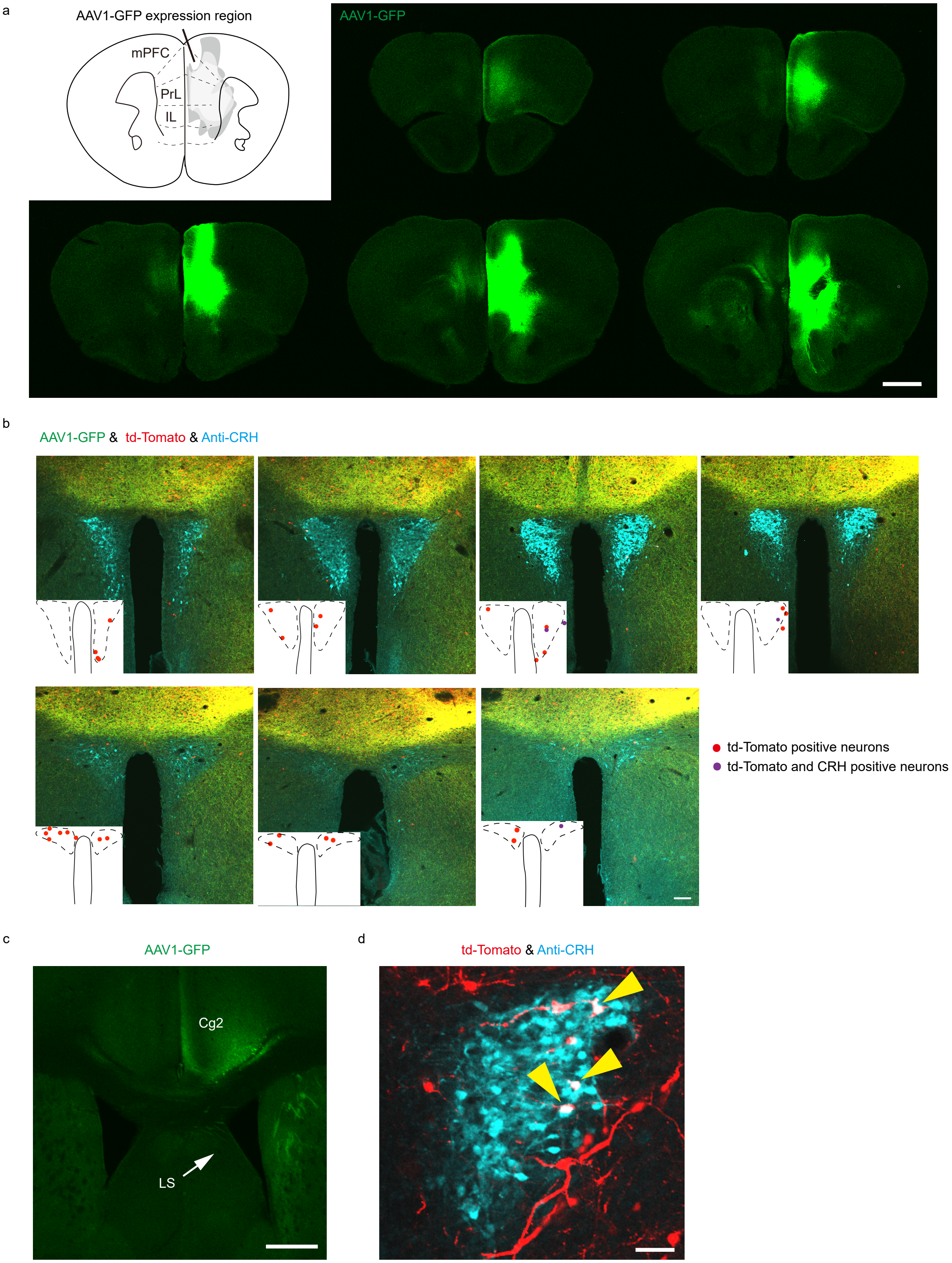

Supplementary Fig. S2. Validation of viral expression in the mPFC and anterograde tracing of mPFC-PVN innervation.

(a) Schematic and representative images showing the AAV1-GFP expression area in the mPFC. **(b)** Representative images and schematic diagrams of mPFC-innervated neurons across the PVN. **(c, d)** A representative sample in which viral expression was restricted to the mPFC without spread to the septal nucleus (c, arrow), and corresponding innervated neurons co-labeled with CRH in the PVN (d, arrowheads). Cg2, cingulate area 2; LS, lateral septum. Scale bars: 1 mm (a), 100 μm (b), 500 μm (c), 50 μm (d).

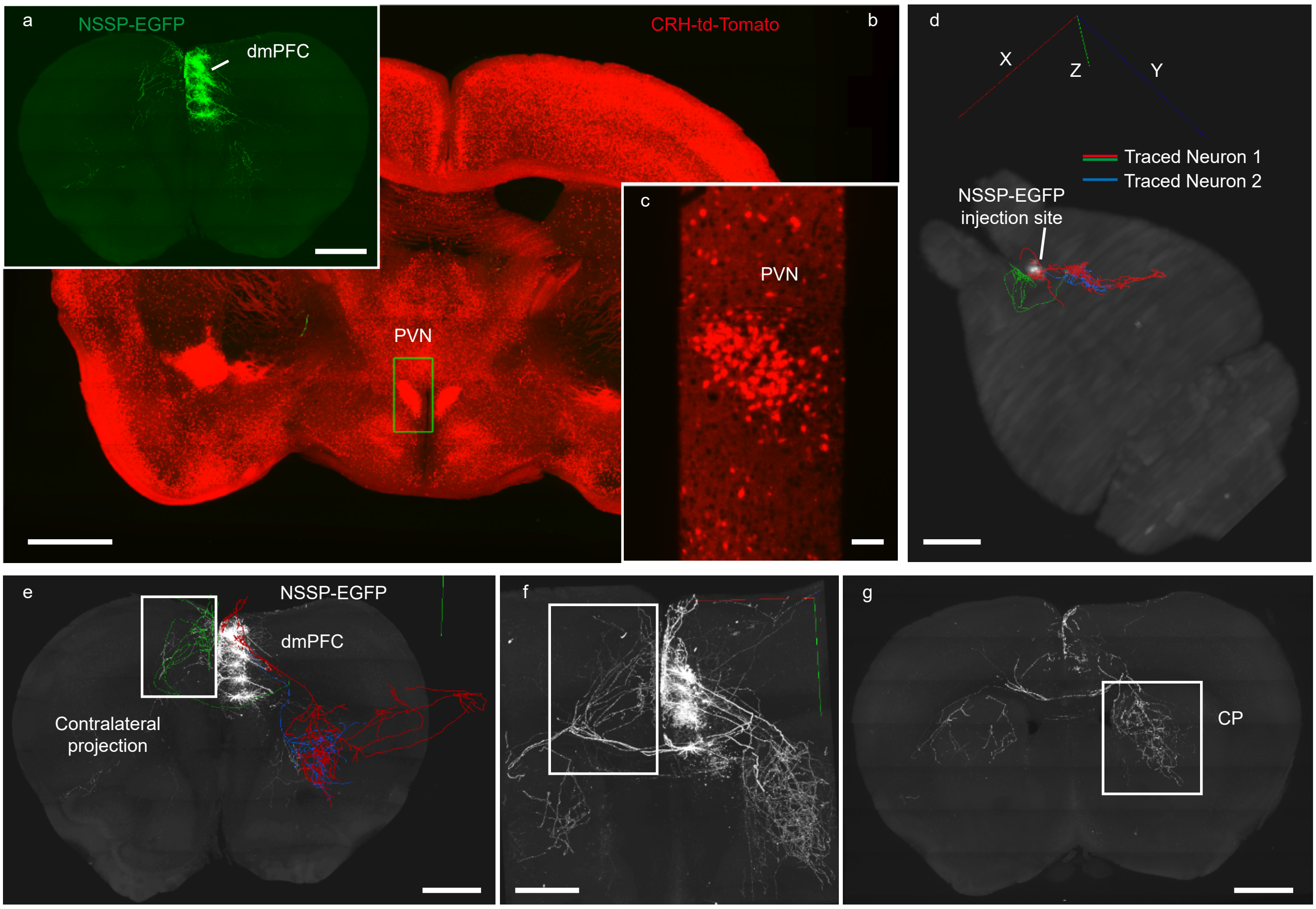

Supplementary Fig. S3. Projection patterns and axonal pathways of NSSP-EGFP-labeled neurons in the dmPFC.

(a–c) EGFP-labeled neurons in the dmPFC (a) and their axonal terminals in the PVN (b, c), (c) shows an enlarged view of the boxed region in (b). **(d–g)** Projection patterns of representative traced neurons originating from the dmPFC. Scale bars: 1 mm (a, b, d, e, g), 50 µm (c), and 500 µm (f).

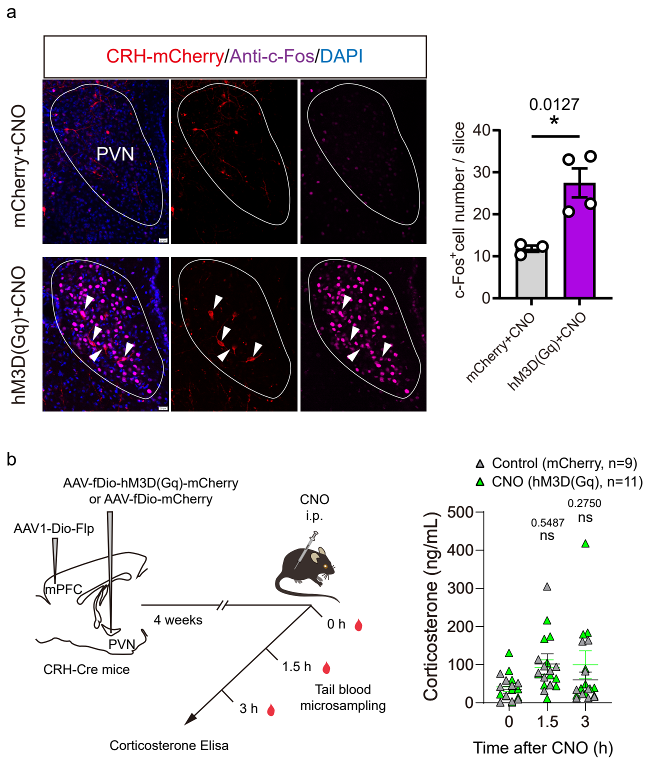

Supplementary Fig. S4. c-Fos expression in the PVN and serum corticosterone levels following chemogenetic activation of the mPFC-PVN circuit in non-stressed mice.
(a) Representative images (Left) and quantitative analysis (right) showing the difference in c-Fos expression in the PVN between the hM3D(Gq) and mCherry control groups. Scale bar = 20 µm; (n = 18 slices from 3 mice for the control group; n = 21 slices from 4 mice for the hM3Dq+CNO group). **(b)** Left: Strategy for circuit activation, blood collection, and corticosterone measurement. Right: Quantitative analysis of serum corticosterone levels at different time points between the hM3D(Gq) and mCherry control groups (n = 9 mice for mCherry group, n = 11 for hM3Dq group). ns, not significant. Data are mean ± SEM. See Supplementary Table S1 for statistical details.

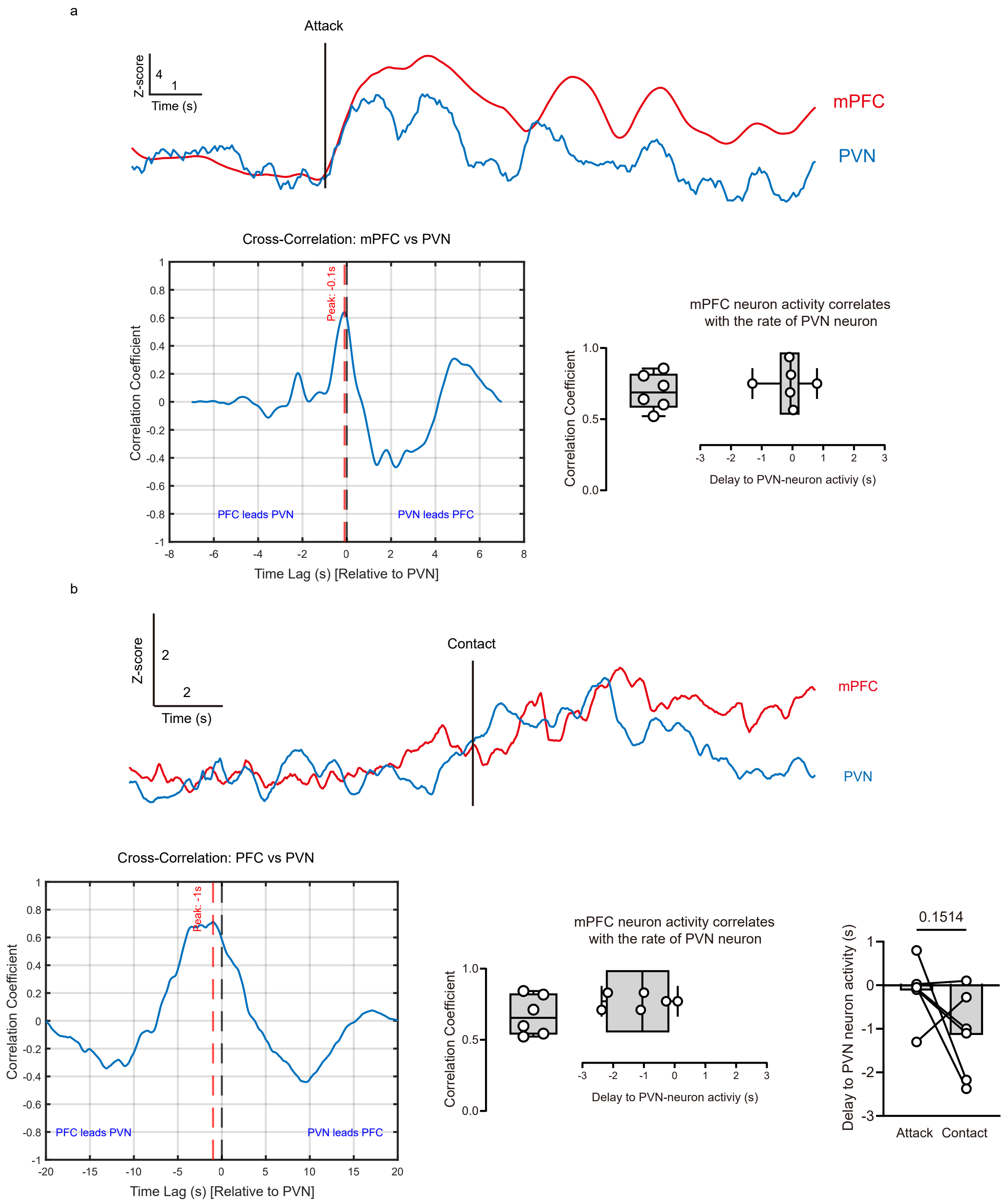

Supplementary Fig. S5. Temporal correlation analysis of activity between mPFC inputs and PVN neurons.

(a) Top: Representative Z-score traces of mPFC input neuron activity (red) and correlated PVN neuron activity (blue) during attack on day 1. Bottom: Peak correlation coefficient and delay of highest correlation in time-lagged cross-correlation between mPFC input and PVN neuron activity during attack (n = 6 mice). **(b)** Top: Representative Z-score traces of mPFC input neuron activity (red) and correlated PVN neuron activity (blue) during social contact on day 2. Bottom: Peak correlation coefficient and delay of highest correlation in time-lagged cross-correlation between mPFC input and PVN neuron activity during contact (n = 6 mice). Data are mean ± SEM. See Supplementary Table S1 for statistical details.

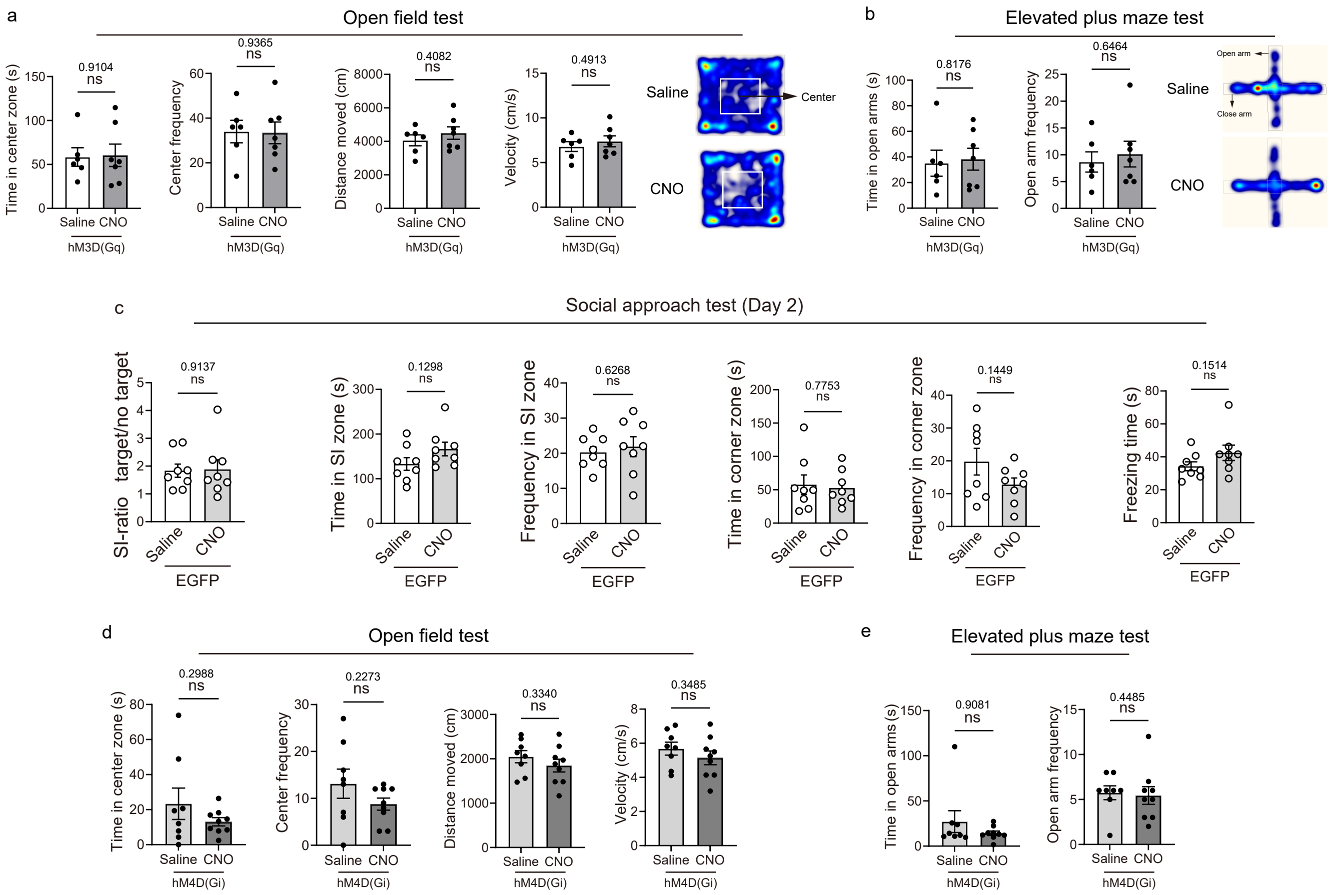

Supplementary Fig. S6. Effects of vmPFC-PVNCRH circuit manipulation on locomotor, anxiety-like behaviors and CNO off-target assessment in mice.

(a, b) Effects of circuit activation on locomotor and anxiety-like behaviors, shown by activity trajectory heatmaps and quantification of central zone time (a) and open arm entries (b) (n = 6 mice for saline group, n = 7 for CNO group). **(c)** Social interaction behaviors in EGFP-control mice treated with saline or CNO, demonstrating no off-target effect of CNO (n = 8 mice per group). **(d, e)** Effects of circuit inhibition on locomotor and anxiety-like behaviors, quantified by central zone time (d) and open arm entries (e) (n = 8 mice for saline group, n = 9 for CNO group). ns, not significant. Data are mean ± SEM. See Supplementary Table S1 for statistical details.

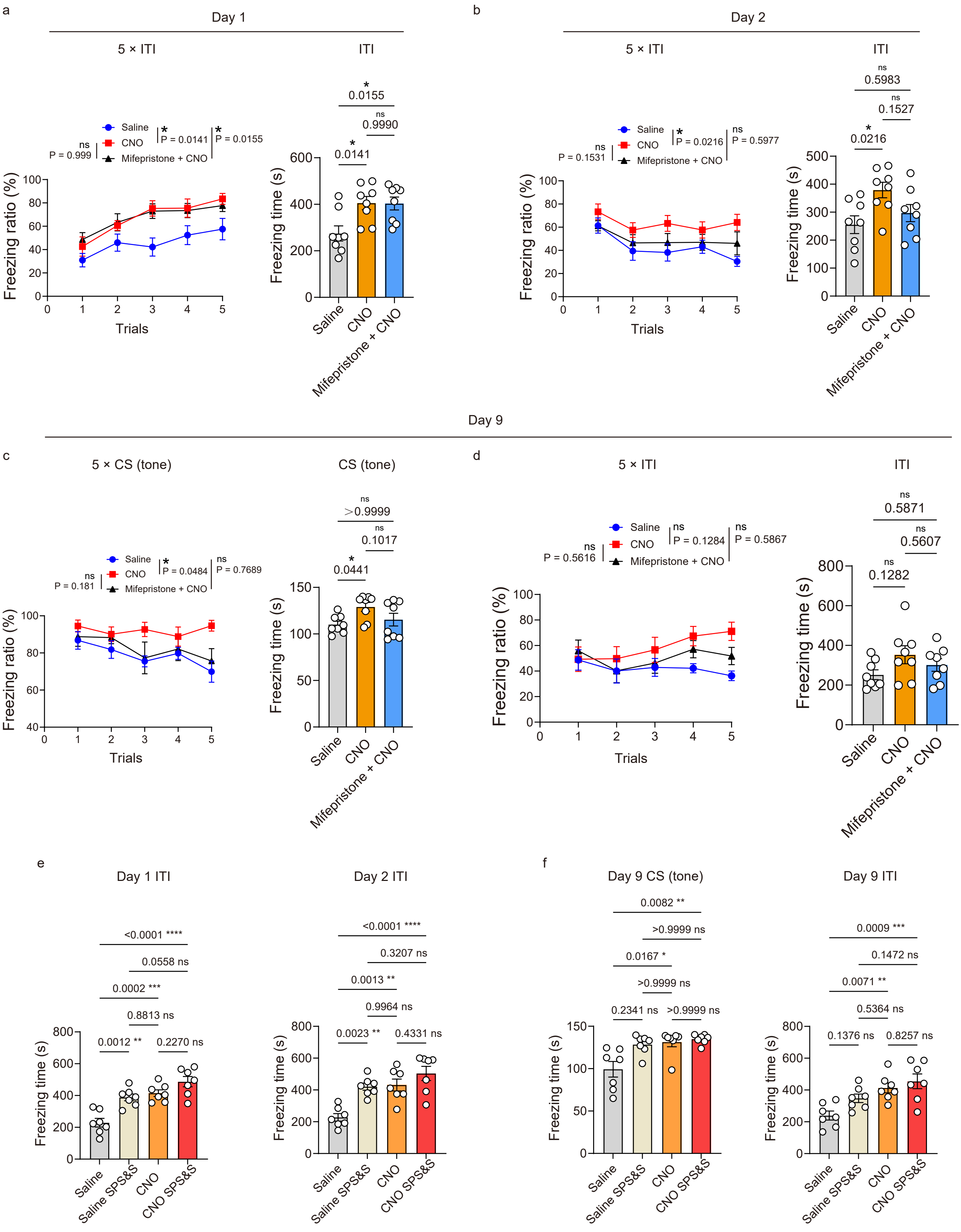

Supplementary Fig. S7. Effect of mifepristone on circuit activation-induced fear behaviors (a–d) and comparison of fear between circuit activation and the SPS&S model (e, f).
(a, b) Freezing during inter trial intervals (ITIs) on day 1 (a) and day 2 (b) in mice treated with saline, CNO, or CNO + mifepristone (n = 8 per group). **(c, d)** Freezing on day 9 during CS (tone) presentation (c) and ITIs (d) in the same treatment groups (n = 8 per group). **(e, f)** Freezing during ITIs on days 1 and 2 (e) and on day 9 during CS (tone) and ITIs (f) across four groups: saline control, SPS&S (Saline SPS&S), circuit activation (CNO), and circuit activation plus SPS&S (CNO SPS&S) (n = 7 per group). ns, not significant. Data are mean ± SEM. See Supplementary Table S1 for statistical details.

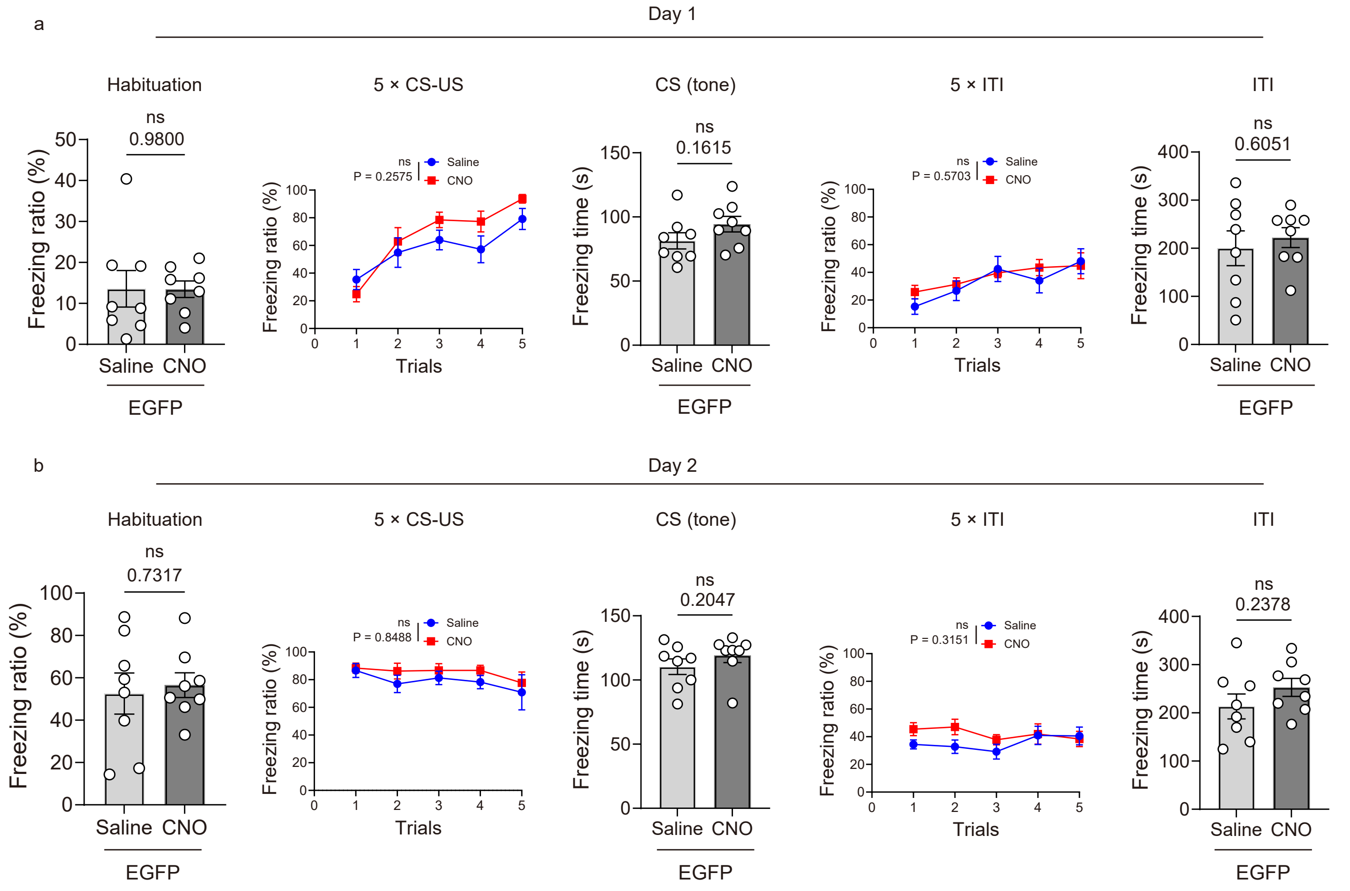

Supplementary Fig. S8. Assessment of potential off-target effects of CNO on fear-related behaviors.

(a, b) Freezing behavior in EGFP-control mice treated with saline or CNO during fear conditioning on day 1 (a) and during extinction recall on day 2 (b) (n = 8 mice per group). ns, not significant. Data are mean ± SEM. See Supplementary Table S1 for statistical details.

Supplementary Table S1. Statistical table.

Statistical table

Data	Response variable	Groups	n define as	Normality Test (Shapiro-Wilk)	Homogeneity of variance test	Hypothesis test	Test value	P value	summary
Figure 2G	Area under cure in calcium recording	Before n=30<br>After n=30	Neuron	W=0.6101 p<0.0001<br>W=0.6101 p<0.0001		Wilcoxon matched-pairs signed-rank test	rs=0.4719 p=0.0042	p<0.0001	****
Figure 2I	AUC	Consistent n=7<br>Cascaded n=7	Pulse	W=0.9116 p=0.4069<br>W=0.9499 p=0.7288	F=182.8 DFn=6 Dfd=6 P<0.0001	Welch's t-test	t=3.778 df=6.066	P=0.0090	**
	Peak	Consistent n=7<br>Cascaded n=7	Pulse	W=0.8978 p=0.3177<br>W=0.8858 p=0.2535	F=2.822 DFn=6 Dfd=6 P=0.2324	Two-tailed unpaired t-test	t=7.555 df=12	p<0.0001	****
	Time to peak	Consistent n=7<br>Cascaded n=7	Pulse	W=0.8616 p=0.1566<br>W=0.9114 p=0.4059	F=15.39 DFn=6 Dfd=6 P=0.0041	Welch's t-test	t=2.986 df=6.776	P=0.0211	*
Figure 2K	Relative mean AUC	5 s duration n=5<br>10 s duration n=5<br>20 s duration n=5<br>30 s duration n=5<br>60 s duration n=5	Mouse	W=0.8425 p=0.1718<br>W=0.9757 p=0.9102<br>W=0.9535 p=0.7620<br>W=0.9051 p=0.4386<br>W=0.9255 p=0.5658		One-way Repeated-Measures ANOVA	F=11.31 DFn=1.520 Dfd=6.080 p=0.0109	Tukey's post hoc test P(5s vs. 10s)=0.9907 P(5s vs. 20s)=0.1753<br>P(5s vs. 30s)=0.0975 P(5s vs. 60s)=0.0052<br>P(10s vs. 20s)=0.1278 P(10s vs. 30s)=0.0425<br>P(10s vs. 60s)=0.0172 P(20s vs. 30s)=0.6849<br>P(20s vs. 60s)=0.4993 P(30s vs. 60s)=0.9195	ns/ns<br>ns/**<br>ns/*<br>*/ns<br>ns/ns
	Time to peak	5 s duration n=5<br>10 s duration n=5<br>20 s duration n=5<br>30 s duration n=5<br>60 s duration n=5	Mouse	W=0.9137 p=0.4901<br>W=0.8413 p=0.1686<br>W=0.9800 p=0.9346<br>W=0.8116 p=0.1004<br>W=0.8374 p=0.1579		One-way Repeated-Measures ANOVA	F=19.27 DFn=1.975 Dfd=7.902 p=0.0009	Tukey's post hoc test P(5s vs. 10s)=0.8270 P(5s vs. 20s)=0.0778<br>P(5s vs. 30s)=0.0200 P(5s vs. 60s)=0.0082<br>P(10s vs. 20s)=0.0038 P(10s vs. 30s)=0.0021<br>P(10s vs. 60s)=0.0679 P(20s vs. 30s)=0.0974<br>P(20s vs. 60s)=0.5933 P(30s vs. 60s)=0.7892	ns/ns<br>*/**<br>**/**<br>ns/ns<br>ns/ns
Figure 2P	AUC per second	Baseline n=7<br>Activation n=7	Neuron	W=0.8871 p=0.2600<br>W=0.8871 p=0.2600		Two-tailed paired t-test	t=3.106 df=6	p=0.0210	*
Figure 2R	Peak frequency	Baseline n=29<br>0-2min after CNO n=29<br>2-4min after CNO n=29	Neuron	W=0.9134 p=0.0209<br>W=0.9015 p=0.0107<br>W=0.9049 p=0.0129		Friedman test	Friedman statistic=37.5 P<0.0001	Dunn's post hoc test P(Baseline vs. 0-2 min after CNO)<0.0001<br>P(Baseline vs. 2-4 min after CNO)=0.0001<br>P(0-2 min after CNO vs. 2-4 min after CNO)=0.2635	****<br>***<br>ns
Figure 2S	AUC per second	Baseline n=54<br>0-2min after CNO n=54<br>2-4min after CNO n=54	Neuron	W=0.6145 p=<0.0001<br>W=0.6742 p=<0.0001<br>W=0.9127 p=<0.0001		Friedman test	Friedman statistic=51.59 P<0.0001	Dunn's post hoc test P(Baseline vs. 0-2 min after CNO)<0.0001<br>P(Baseline vs. 2-4 min after CNO)<0.0001<br>P(0-2 min after CNO vs. 2-4 min after CNO)=0.0807	****<br>****<br>ns
Figure 2U	c-fos+ GCaMP+ / GCaMP+ (%)	mCherry + CNO n=4<br>hM3D(Gq) + CNO n=4	Mouse	W=0.8391 p=0.1928<br>W=0.9102 p=0.4835	F=1.847 DFn=3 Dfd=3 P=0.6268	Two-tailed unpaired t-test	t=5.500 df=6	P=0.0015	**
Figure 2V	Corticosterone lever	mCherry no FS n=8<br>mCherry FS n=8<br>hM3D(Gq) FS n=8<br>hM4D(Gi) FS n=8	Mouse	W=0.9143 p=0.3856<br>W=0.9234 p=0.4578<br>W=0.9141 p=0.3841<br>W=0.8683 p=0.1450	F=6.959 DFn=3 Dfd=32 P=0.0010	Welch's ANOVA test	W=59.31 DFn=3 Dfd=12.56 P<0.0001	Dunnett's T3 post hoc test P(mCherry no FS vs. mCherry FS)=0.0002<br>P(mCherry no FS vs. hM3D(Gq) FS)=0.0003 P(mCherry no FS vs. hM4D(Gi) FS)=0.0002<br>P(mCherry FS vs. hM3D(Gq) FS)=0.0043 P(mCherry FS vs. hM4D(Gi) FS)=0.2709<br>P(hM3D(Gq) FS vs. hM4D(Gi) FS)=0.0298	***<br>***/***<br>**/ns<br>*
Figure 3D	Day 1 First attack Z-score (mPFC inputs)	Before n=6<br>Attack n=6	Mouse	W=0.9020 p=0.3857<br>W=0.9020 p=0.3857		Two-tailed paired t-test	t=3.582 df=5	P=0.0158	*
	Day 1 First attack Z-score (PVN neurons)	Before n=6<br>Attack n=6	Mouse	W=0.8612 p=0.1935<br>W=0.8612 p=0.1935		Two-tailed paired t-test	t=2.798 df=5	p=0.0381	*
Figure 3E	Day 1 Attack Z-score (mPFC inputs)	Before n=6<br>Attack n=6	Mouse	W=0.9173 p=0.4863<br>W=0.9173 p=0.4863		Two-tailed paired t-test	t=4.36 df=5	P=0.0073	**
	Day 1 Attack Z-score (PVN neurons)	Before n=6<br>Attack n=6	Mouse	W=0.8448 p=0.1429<br>W=0.8448 p=0.1429		Two-tailed paired t-test	t=3.051 df=5	P=0.0284	*
Figure 3F	Day 2 First contact Z-score (mPFC inputs)	Before n=6<br>Contact n=6	Mouse	W=0.7441 p=0.0174<br>W=0.7441 p=0.0174		Wilcoxon matched-pairs signed-rank test	rs=-0.6000 p=0.1208	P=0.5625	ns
	Day 2 First contact Z-score (PVN neurons)	Before n=6<br>Contact n=6	Mouse	W=0.9206 p=0.5096<br>W=0.9206 p=0.5096		Two-tailed paired t-test	t=3.874 df=5	P=0.0117	*
Figure 3G	Day 2 in corner Z-score (mPFC inputs)	Before n=14<br>Corner n=14	Event	W=0.7740 p=0.0024<br>W=0.7740 p=0.0024		Wilcoxon matched-pairs signed-rank test	rs=0.3714 p=0.0959	P=0.0001	***
	Day 2 in corner Z-score (PVN neurons)	Before n=14<br>Corner n=14	Event	W=0.8676 p=0.0388<br>W=0.8676 p=0.0388		Wilcoxon matched-pairs signed-rank test	rs=0.5516 p=0.0219	P=0.0580	ns
Figure 3H	Day 2 multiple contacts Z-score (mPFC inputs)	Before n=6<br>Contact n=6	Mouse	W=0.8301 p=0.1078<br>W=0.8301 p=0.1078		Two-tailed paired t-test	t=3.230 df=5	P=0.0232	*
	Day 2 multiple contacts Z-score (PVN neurons)	Before n=6<br>Contact n=6	Mouse	W=0.9771 p=0.9364<br>W=0.9771 p=0.9364		Two-tailed paired t-test	t=4.788 df=5	P=0.0049	**
Figure 3K	Social interaction ratio (Control vs. Chronic social defeat)	Control n=6<br>CSD n=5	Mouse	W=0.9547 p=0.7780<br>W=0.8479 p=0.1880	F=2.430 DFn=5 Dfd=4 P=0.4102	Two-tailed unpaired t-test	t=2.329 df=9	P=0.0448	*
	Immobility time in forced swim test (Control vs. Chronic social defeat)	Control n=6<br>CSD n=5	Mouse	W=0.8147 p=0.0794<br>W=0.9107 p=0.4720	F=2.319 DFn=4 Dfd=5 P=0.3810	Two-tailed unpaired t-test	t=2.416 df=9	P=0.0389	*
Figure 3M	% of FosB expression in RV-labeled cells in mPFC	Control n=3<br>CSD n=3	Mouse	W=0.9981 p=0.9178<br>W=0.8797 p=0.3236	F=2.002 DFn=2 Dfd=2 P=0.6662	Two-tailed unpaired t-test	t=3.061 df=4	P=0.0376	*
Figure 4D	Day 2 SI-ratio (saline vs. CNO circuit activation)	Saline n=8<br>CNO n=7	Mouse	W=0.9044 p=0.3164<br>W=0.9147 p=0.4291	F=5.837 DFn=6 Dfd=7 P=0.0354	Two-tailed unpaired t-test, Welch's correction	t=2.375 df=7.784	P=0.0457	*
Figure 4E	Day 2 Time in SI zone (saline vs. CNO circuit activation)	Saline n=8<br>CNO n=7	Mouse	W=0.9036 p=0.3112<br>W=0.9531 p=0.7575	F=3.920 DFn=7 Dfd=6 P=0.1166	Two-tailed unpaired t-test	t=8.578 df=13	P<0.0001	****
	Day 2 Frequency in SI zone (saline vs. CNO circuit activation)	Saline n=8<br>CNO n=7	Mouse	W=0.9215 p=0.4418<br>W=0.9161 p=0.4396	F=1.241 DFn=7 Dfd=6 P=0.8085	Two-tailed unpaired t-test	t=1.890 df=13	P=0.0813	ns
	Day 2 Time in corner zone (saline vs. CNO circuit activation)	Saline n=8<br>CNO n=7	Mouse	W=0.8850 p=0.2099<br>W=0.8640 p=0.1644	F=5.517 DFn=6 Dfd=7 P=0.0411	Two-tailed unpaired t-test, Welch's correction	t=4.623 df=7.884	P=0.0018	**
	Day 2 Frequency in corner zone (saline vs. CNO circuit activation)	Saline n=8<br>CNO n=7	Mouse	W=0.9384 p=0.5955<br>W=0.9767 p=0.9420	F=1.696 DFn=6 Dfd=7 P=0.5044	Two-tailed unpaired t-test	t=3.870 df=13	P=0.0019	**
Figure 4G	Day 7 Time in SI zone (saline vs. CNO circuit activation)	Saline n=8<br>CNO n=7	Mouse	W=0.8590 p=0.1172<br>W=0.8555 p=0.1378	F=1.942 DFn=6 Dfd=7 P=0.4052	Two-tailed unpaired t-test	t=1.463 df=13	P=0.1673	ns
	Day 7 Frequency in SI zone (saline vs. CNO circuit activation)	Saline n=8<br>CNO n=7	Mouse	W=0.9697 p=0.8957<br>W=0.9433 p=0.6690	F=2.570 DFn=7 Dfd=6 P=0.2705	Two-tailed unpaired t-test	t=5.376 df=13	P=0.0001	***
	Day 7 Time in corner zone (saline vs. CNO circuit activation)	Saline n=8<br>CNO n=7	Mouse	W=0.8774 p=0.1778<br>W=0.9509 p=0.7380	F=3.382 DFn=6 Dfd=7 P=0.1360	Two-tailed unpaired t-test	t=2.216 df=13	P=0.0451	*
	Day 7 Frequency in corner zone (saline vs. CNO circuit activation)	Saline n=8<br>CNO n=7	Mouse	W=0.8651 p=0.1348<br>W=0.9073 p=0.3772	F=1.056 DFn=7 Dfd=6 P=0.9632	Two-tailed unpaired t-test	t=0.3276 df=13	P=0.7484	ns
Figure 4H	Day 2 Freezing time (saline vs. CNO circuit activation)	Saline n=8<br>CNO n=7	Mouse	W=0.9107 p=0.3592<br>W=0.9644 p=0.8558	F=1.970 DFn=6 Dfd=7 P=0.3958	Two-tailed unpaired t-test	t=2.616 df=13	P=0.0214	*
Figure 4I	Day 30 Time in SI zone (saline vs. CNO circuit activation)	Saline n=8<br>CNO n=7	Mouse	W=0.9024 p=0.3038<br>W=0.8396 p=0.0986	F=7.822 DFn=6 Dfd=7 P=0.0158	Two-tailed unpaired t-test, Welch's correction	t=1.951 df=7.339	P=0.0901	ns
	Day 30 Frequency in SI zone (saline vs. CNO circuit activation)	Saline n=8<br>CNO n=7	Mouse	W=0.9658 p=0.8636<br>W=0.9051 p=0.3630	F=1.772 DFn=7 Dfd=6 P=0.5030	Two-tailed unpaired t-test	t=3.373 df=13	P=0.0050	**
	Day 30 Time in corner zone (saline vs. CNO circuit activation)	Saline n=8<br>CNO n=7	Mouse	W=0.9559 p=0.7701<br>W=0.8878 p=0.2635	F=36.10 DFn=6 Dfd=7 P=0.0001	Two-tailed unpaired t-test, Welch's correction	t=2.090 df=6.291	P=0.0795	ns
	Day 30 Frequency in corner zone (saline vs. CNO circuit activation)	Saline n=8<br>CNO n=7	Mouse	W=0.9143 p=0.3856<br>W=0.6452 p=0.0009	F=17.53 DFn=6 Dfd=7 P=0.0014	Mann-Whitney U test	U=25.50	P=0.8019	ns
Figure 4J	Day 30 Freezing time (saline vs. CNO circuit activation)	Saline n=8<br>CNO n=7	Mouse	W=0.8013 p=0.0296<br>W=0.9474 p=0.7058	F=1.055 DFn=7 Dfd=6 P=0.9644	Mann-Whitney U test	U=22	P=0.5358	ns
Figure 4N	Day 2 SI-ratio (saline vs. CNO circuit inhibition)	Saline n=8<br>CNO n=9	Mouse	W=0.9279 p=0.4973<br>W=0.9419 p=0.6017	F=9.514 DFn=8 Dfd=7 P=0.0076	Two-tailed unpaired t-test, Welch's correction	t=2.987 df=9.846	P=0.0139	*
Figure 4O	Day 2 Time in SI zone (saline vs. CNO circuit inhibition)	Saline n=8<br>CNO n=9	Mouse	W=0.9319 p=0.5331<br>W=0.8817 p=0.1635	F=20.40 DFn=8 Dfd=7 P=0.0007	Two-tailed unpaired t-test, Welch's correction	t=2.605 df=8.876	P=0.0288	*
	Day 2 Frequency in SI zone (saline vs. CNO circuit inhibition)	Saline n=8<br>CNO n=9	Mouse	W=0.7990 p=0.0279<br>W=0.9127 p=0.3353	F=2.521 DFn=8 Dfd=7 P=0.2403	Mann-Whitney U test	U=11.50	P=0.0148	*
	Day 2 Time in corner zone (saline vs. CNO circuit inhibition)	Saline n=8<br>CNO n=9	Mouse	W=0.9273 p=0.4921<br>W=0.9165 p=0.3642	F=1.428 DFn=7 Dfd=8 P=0.6257	Two-tailed unpaired t-test	t=1.570 df=15	P=0.1373	ns
	Day 2 Frequency in corner zone (saline vs. CNO circuit inhibition)	Saline n=8<br>CNO n=9	Mouse	W=0.9459 p=0.6702<br>W=0.9576 p=0.7733	F=1.023 DFn=8 Dfd=7 P=0.9891	Two-tailed unpaired t-test	t=1.388 df=15	P=0.1855	ns
	Day 2 Velocity in SI zone (saline vs. CNO circuit inhibition)	Saline n=5<br>CNO n=7	Mouse	W=0.7945 p=0.0730<br>W=0.9430 p=0.6656	F=5.433 DFn=6 Dfd=4 P=0.1233	Two-tailed unpaired t-test	t=0.1465 df=10	P=0.8865	ns
Figure 4P	Day 2 Freezing time (saline vs. CNO circuit inhibition)	Saline n=8<br>CNO n=9	Mouse			Two-way ANOVA	F (Behavior × Gene/pharmacology)=5.706 DFn=1 Dfd=15 P=0.0305<br>F (Gene/pharmacology)=5.396 DFn=1 Dfd=15 P=0.0346	Bonferroni's post hoc test P (No target Saline vs. CNO)=0.4880<br>P (target Saline vs. CNO)=0.0088	ns<br>**
Figure 5B	Day 1 habituation freezing ratio (saline vs. CNO circuit activation)	Saline n=7<br>CNO n=7	Mouse	W=0.9056 p=0.3664<br>W=0.9260 p=0.5171	F=1.759 DFn=6 Dfd=6 P=0.5097	Two-tailed unpaired t-test	t=0.9852 df=12	P=0.3440	ns
Figure 5C	Day 1 5 x CS-US freezing ratio (saline vs. CNO circuit activation)	Saline n=7<br>CNO n=7	Mouse			Two-way ANOVA	F (Trial × Gene/pharmacology)=3.711 DFn=4 Dfd=48 P=0.0104<br>F (Trial)=31.28 DFn=4 Dfd=48 P<0.0001<br>F (Gene/pharmacology)=0.2447 DFn=1 Dfd=12 P=0.6298	Tukey's post hoc test P(trial1 Saline vs. CNO)=0.7070<br>P(trial2 Saline vs. CNO)=0.0091 P(trial3 Saline vs. CNO)=0.6830<br>P(trial4 Saline vs. CNO)=0.5914 P(trial5 Saline vs. CNO)=0.1467	ns<br>**/ns<br>ns/ns
Figure 5D	Day 1 CS (tone) freezing time (saline vs. CNO circuit activation)	Saline n=7<br>CNO n=7	Mouse	W=0.8742 p=0.2019<br>W=0.9563 p=0.7867	F=1.651 DFn=6 Dfd=6 P=0.5575	Two-tailed unpaired t-test	t=0.4985 df=12	P=0.6272	
Figure 5E	Day 1 5 x ITI freezing ratio (saline vs. CNO circuit activation)	Saline n=7<br>CNO n=7	Mouse			Two-way ANOVA	F (Trial × Gene/pharmacology)=0.8235 DFn=3.050 Dfd=36.59 P=0.4911<br>F (Trial)=6.750 DFn=3.050 Dfd=36.59 P=0.0009<br>F (Gene/pharmacology)=4.899 DFn=1 Dfd=12 P=0.0470	Tukey's post hoc test P(trial1 Saline vs. CNO)=0.2161<br>P(trial2 Saline vs. CNO)=0.0075 P(trial3 Saline vs. CNO)=0.0887<br>P(trial4 Saline vs. CNO)=0.3012 P(trial5 Saline vs. CNO)=0.1692	ns<br>**/ns<br>ns/ns
Figure 5F	Day 1 ITI freezing time (saline vs. CNO circuit activation)	Saline n=7<br>CNO n=7	Mouse	W=0.9136 p=0.4212<br>W=0.9460 p=0.6930	F=1.306 DFn=6 Dfd=6 P=0.7539	Two-tailed unpaired t-test	t=2.212 df=12	P=0.0471	*
Figure 5G	Day 2 habituation freezing ratio (saline vs. CNO circuit activation)	Saline n=7<br>CNO n=7	Mouse	W=0.8795 p=0.2241<br>W=0.9754 p=0.9345	F=1.018 DFn=6 Dfd=6 P=0.9830	Two-tailed unpaired t-test	t=0.8053 df=12	P=0.4363	ns
Figure 5H	Day 2 5 x CS (tone) freezing ratio (saline vs. CNO circuit activation)	Saline n=7<br>CNO n=7	Mouse			Two-way ANOVA	F (Trial × Gene/pharmacology)=0.7675 DFn=2.641 Dfd=31.7 P=0.5058<br>F (Trial)=1.199 DFn=2.641 Dfd=31.70 P=0.3231<br>F (Gene/pharmacology)=1.395 DFn=1 Dfd=12 P=0.2604	Tukey's post hoc test P(trial1 Saline vs. CNO)=0.7358<br>P(trial2 Saline vs. CNO)=0.7500 P(trial3 Saline vs. CNO)=0.4771<br>P(trial4 Saline vs. CNO)=0.0529 P(trial5 Saline vs. CNO)=0.1618	ns
Figure 5I	Day 2 CS (tone) freezing time (saline vs. CNO circuit activation)	Saline n=7<br>CNO n=7	Mouse	W=0.9823 p=0.9698<br>W=0.8764 p=0.2111	F=2.024 DFn=6 Dfd=6 P=0.4120	Two-tailed unpaired t-test	t=1.177 df=12	P=0.2622	ns
Figure 5J	Day 2 5 x ITI freezing ratio (saline vs. CNO circuit activation)	Saline n=7<br>CNO n=7	Mouse			Two-way ANOVA	F (Trial × Gene/pharmacology)=0.5202 DFn=1.984 Dfd=23.81 P=0.5996<br>F (Trial)=0.8785 DFn=1.984 Dfd=23.81 P=0.4277<br>F (Gene/pharmacology)=9.405 DFn=1 Dfd=12 P=0.0098	Tukey's post hoc test P(trial1 Saline vs. CNO)=0.0231<br>P(trial2 Saline vs. CNO)=0.0149 P(trial3 Saline vs. CNO)=0.0230<br>P(trial4 Saline vs. CNO)=0.0188 P(trial5 Saline vs. CNO)=0.0267	*<br>*/*<br>*/*
Figure 5K	Day 2 ITI freezing time (saline vs. CNO circuit activation)	Saline n=7<br>CNO n=7	Mouse	W=0.7575 p=0.0152<br>W=0.8593 p=0.1493	F=1.021 DFn=6 Dfd=6 P=0.9808	Mann-Whitney U test	U=8	P=0.0379	*
Figure 5L	Day 9 habituation freezing ratio (saline vs. CNO circuit activation)	Saline n=7<br>CNO n=7	Mouse	W=0.7655 p=0.0184<br>W=0.9220 p=0.4854	F=1.656 DFn=6 Dfd=6 P=0.5556	Mann-Whitney U test	U=17	P=0.3829	ns
Figure 5M	Day 9 5 x CS (tone) freezing ratio (saline vs. CNO circuit activation)	Saline n=7<br>CNO n=7	Mouse			Two-way ANOVA	F (Trial × Gene/pharmacology)=5.561 DFn=4 Dfd=48 P=0.0009<br>F (Trial)=1.434 DFn=2.567 Dfd=30.91 P=0.2534<br>F (Gene/pharmacology)=15.53 DFn=1 Dfd=12 P=0.0020	Tukey's post hoc test P(trial1 Saline vs. CNO)=0.1016<br>P(trial2 Saline vs. CNO)=0.0286 P(trial3 Saline vs. CNO)=0.0196<br>P(trial4 Saline vs. CNO)=0.0011 P(trial5 Saline vs. CNO)=0.0061	ns<br>*/*<br>**/**
Figure 5N	Day 9 CS (tone) freezing time (saline vs. CNO circuit activation)	Saline n=7<br>CNO n=7	Mouse	W=0.8614 p=0.1557<br>W=0.8176 p=0.0609	F=3.804 DFn=6 Dfd=6 P=0.1287	Two-tailed unpaired t-test	t=3.940 df=12	P=0.0020	**
Figure 5O	Day 9 5 x ITI freezing ratio (saline vs. CNO circuit activation)	Saline n=7<br>CNO n=7	Mouse			Two-way ANOVA	F (Trial × Gene/pharmacology)=1.366 DFn=4 Dfd=48 P=0.2596<br>F (Trial)=2.564 DFn=3.034 Dfd=36.41 P=0.0690<br>F (Gene/pharmacology)=4.510 DFn=1 Dfd=12 P=0.0552	Tukey's post hoc test P(trial1 Saline vs. CNO)=0.0972<br>P(trial2 Saline vs. CNO)=0.0887 P(trial3 Saline vs. CNO)=0.0705<br>P(trial4 Saline vs. CNO)=0.0272 P(trial5 Saline vs. CNO)=0.3646	ns<br>ns/ns<br>*/ns
Figure 5P	Day 9 ITI freezing time (saline vs. CNO circuit activation)	Saline n=7<br>CNO n=7	Mouse	W=0.7691 p=0.0200<br>W=0.9527 p=0.7545	F=1.772 DFn=6 Dfd=6 P=0.5041	Mann-Whitney U test	U=10	P=0.0728	ns
Figure 5Q	Comparison of CS (tone) freezing time of day1/2/9 in saline group	Day1 n=7<br>Day2 n=7<br>Day9 n=7	Mouse	W=0.9572 p=0.7942<br>W=0.9080 p=0.3819<br>W=0.7745 p=0.0227		Friedman test	Friedman statistic=10.57 P=0.0027	Tukey's post hoc test P(Day1 vs Day2)=0.0226<br>P(Day1 vs Day9)>0.9999<br>P(Day2 vs Day9)=0.0099	*<br>ns<br>**
	Comparison of CS (tone) freezing time of day1/2/9 in CNO group	Day1 n=7<br>Day2 n=7<br>Day9 n=7	Mouse	W=0.9840 p=0.9765<br>W=0.8772 p=0.2142<br>W=0.9069 p=0.3749		One-way Repeated-Measures ANOVA	F=14.30 DFn=1.299 Dfd=7.792 P=0.0041	Tukey's post hoc test P(Day1 vs Day2)=0.0016<br>P(Day1 vs Day9)=0.0373<br>P(Day2 vs Day9)=0.8181	**<br>*<br>ns
Figure 5R	Comparison of ITI freezing time of day1/2/9 in saline group	Day1 n=7<br>Day2 n=7<br>Day9 n=7	Mouse	W=0.9605 p=0.8227<br>W=0.8899 p=0.2741<br>W=0.9656 p=0.8647		One-way Repeated-Measures ANOVA	F=0.4940 DFn=1.289 Dfd=7.736 P=0.5493	Tukey's post hoc test P(Day1 vs Day2)=0.8975<br>P(Day1 vs Day9)=0.6941<br>P(Day2 vs Day9)=0.6396	ns
	Comparison of ITIfreezing time of day1/2/9 in CNO group	Day1 n=7<br>Day2 n=7<br>Day9 n=7	Mouse	W=0.9190 p=0.4613<br>W=0.9001 p=0.3317<br>W=0.9832 p=0.9736		One-way Repeated-Measures ANOVA	F=3.649 DFn=1.740 Dfd=10.44 P=0.0674	Tukey's post hoc test P(Day1 vs Day2)=0.2340<br>P(Day1 vs Day9)=0.5225<br>P(Day2 vs Day9)=0.1357	ns
Figure 5T	Day 1 5 x CS-US freezing ratio (saline vs. CNO circuit inhibition)	Saline n=6<br>CNO n=9	Mouse			Two-way ANOVA	F (Trial × Gene/pharmacology)=1.206 DFn=3.233 Dfd=42.03 P=0.3205<br>F (Trial)=24.23 DFn=3.233 Dfd=42.03 P<0.0001<br>F (Gene/pharmacology)=0.3373 DFn=1 Dfd=13 P=0.5713	Tukey's post hoc test P(trial1 Saline vs. CNO)=0.9908<br>P(trial2 Saline vs. CNO)=0.4666 P(trial3 Saline vs. CNO)=0.2382<br>P(trial4 Saline vs. CNO)=0.4126 P(trial5 Saline vs. CNO)=0.4600	ns
	Day 1 5 x ITI freezing ratio (saline vs. CNO circuit inhibition)	Saline n=6<br>CNO n=9	Mouse			Two-way ANOVA	F (Trial × Gene/pharmacology)=0.3046 DFn=3.015 Dfd=39.19 P=0.8228<br>F (Trial)=12.87 DFn=3.015 Dfd=39.19 P<0.0001<br>F (Gene/pharmacology)=0.008523 DFn=1 Dfd=13 P=0.9279	Tukey's post hoc test P(trial1 Saline vs. CNO)=0.6590<br>P(trial2 Saline vs. CNO)=0.7450 P(trial3 Saline vs. CNO)=0.9748<br>P(trial4 Saline vs. CNO)=0.8648 P(trial5 Saline vs. CNO)=0.7288	ns
Figure 5U	Day 1 habituation freezing ratio (saline vs. CNO circuit inhibition)	Saline n=6<br>CNO n=9	Mouse	W=0.9120 p=0.4497<br>W=0.8719 p=0.1287	F=3.433 DFn=5 Dfd=8 P=0.1189	Two-tailed unpaired t-test	t=2.038 df=13	P=0.0624	ns
	Day 1 CS (tone) freezing time (saline vs. CNO circuit inhibition)	Saline n=6<br>CNO n=9	Mouse	W=0.9700 p=0.8923<br>W=0.8911 p=0.2050	F=1.069 DFn=5 Dfd=8 P=0.8862	Two-tailed unpaired t-test	t=0.5793 df=13	P=0.5723	ns
	Day 1 ITI freezing time (saline vs. CNO circuit inhibition)	Saline n=6<br>CNO n=9	Mouse	W=0.9196 p=0.5023<br>W=0.9562 p=0.7584	F=1.510 DFn=5 Dfd=8 P=0.5749	Two-tailed unpaired t-test	t=0.2905 df=13	P=0.7760	ns
Figure 5V	Day 2 5 x CS (tone) freezing ratio (saline vs. CNO circuit inhibition)	Saline n=6<br>CNO n=9	Mouse			Two-way ANOVA	F (Trial × Gene/pharmacology)=0.2583 DFn=2.384 Dfd=30.99 P=0.8106<br>F (Trial)=2.785 DFn=2.384 Dfd=30.99 P=0.0686<br>F (Gene/pharmacology)=0.09227 DFn=1 Dfd=13 P=0.7661	Tukey's post hoc test P(trial1 Saline vs. CNO)=0.5137<br>P(trial2 Saline vs. CNO)=0.6245 P(trial3 Saline vs. CNO)=0.7242<br>P(trial4 Saline vs. CNO)=0.8750 P(trial5 Saline vs. CNO)=0.8900	ns
	Day 2 5 x ITI freezing ratio (saline vs. CNO circuit inhibition)	Saline n=6<br>CNO n=9	Mouse			Two-way ANOVA	F (Trial × Gene/pharmacology)=0.1835 DFn=3.124 Dfd=40.61 P=0.9132<br>F (Trial)=4.195 DFn=3.124 Dfd=40.61 P=0.0104<br>F (Gene/pharmacology)=0.4899 DFn=1 Dfd=13 P=0.4963	Tukey's post hoc test P(trial1 Saline vs. CNO)=0.6631<br>P(trial2 Saline vs. CNO)=0.8219 P(trial3 Saline vs. CNO)=0.4200<br>P(trial4 Saline vs. CNO)=0.7479 P(trial5 Saline vs. CNO)=0.3964	ns
Figure 5W	Day 2 habituation freezing ratio (saline vs. CNO circuit inhibition)	Saline n=6<br>CNO n=9	Mouse	W=0.8942 p=0.3408<br>W=0.9880 p=0.9928	F=1.080 DFn=8 Dfd=5 P=0.9770	Two-tailed unpaired t-test	t=0.4599 df=13	P=0.6532	ns
	Day 2 CS (tone) freezing time (saline vs. CNO circuit inhibition)	Saline n=6<br>CNO n=9	Mouse	W=0.5860 p=0.0003<br>W=0.7963 p=0.0185	F=6.381 DFn=5 Dfd=8 P=0.0224	Mann-Whitney U test	U=15	P=0.1810	ns
	Day 2 ITI freezing time (saline vs. CNO circuit inhibition)	Saline n=6<br>CNO n=9	Mouse	W=0.9418 p=0.6739<br>W=0.9379 p=0.5601	F=1.847 DFn=8 Dfd=5 P=0.5175	Two-tailed unpaired t-test	t=0.6993 df=13	P=0.4967	ns
Figure 5X	Comparison of CS (tone) freezing time of day1/2 in saline group	Day1 n=6<br>Day2 n=6	Mouse	W=0.9841 p=0.9701<br>W=0.9841 p=0.9701		Two-tailed paired t-test	t=3.377 df=5	P=0.0197	*
	Comparison of CS (tone) freezing time of day1/2 in CNO group	Day1 n=9<br>Day2 n=9	Mouse	W=0.8630 p=0.1033<br>W=0.8630 p=0.1033		Two-tailed paired t-test	t=7.308 df=8	P<0.0001	****
Figure 5Y	Comparison of ITI freezing time of day1/2 in saline group	Day1 n=6<br>Day2 n=6	Mouse	W=0.9038 p=0.3969<br>W=0.9038 p=0.3969		Two-tailed paired t-test	t=0.7689 df=5	P=0.4767	ns
	Comparison of ITI freezing time of day1/2 in CNO group	Day1 n=9<br>Day2 n=9	Mouse	W=0.9764 p=0.9431<br>W=0.9764 p=0.9431		Two-tailed paired t-test	t=2.204 df=8	P=0.0587	ns
Figure S4A	C-Fos+cell number in PVN (mCherry+CNO vs. hM3Dq+CNO)	mCherry + CNO n=3<br>hM3D(Gq) + CNO n=4	Mouse	W=0.8929 p=0.3631<br>W=0.8210 p=0.1456	F=27.15 DFn=3 Dfd=2 P=0.0715	Two-tailed unpaired t-test	t=3.797 df=5	P=0.0127	*
Figure S4B	Corticosterone lever	mCherry + CNO n=9<br>hM3D(Gq) + CNO n=11	Mouse			Two-way ANOVA	F (Time × Gene)=0.9136 DFn=1.619 Dfd=29.15 P=0.3933<br>F (Time)=2.559 DFn=1.619 Dfd=29.15 P=0.1042<br>F (Gene)=0.5734 DFn=1 Dfd=18 P=0.4587	Tukey's post hoc test P(time 0 mCherry vs. hM3Dq)=0.5423<br>P(time 1.5 mCherry vs. hM3Dq)=0.5487 P(time 3 mCherry vs. hM3Dq)=0.2750<br>P(mCherry time 0 vs. 1.5)=0.1348 P(mCherry time 0 vs. 3)=0.4192<br>P(mCherry time 1.5 vs. 3)=0.5063 P(hM3Dq time 0 vs. 1.5)=0.3582<br>P(hM3Dq time 0 vs. 3)=0.2997 P(hM3Dq time 1.5 vs. 3)=0.8468	ns
Figure S5B	Delay to PVN neuron activity (attack vs. contact)	Attack n=6<br>Contact n=6	Mouse	W=0.8492 p=0.1551<br>W=0.9283 p=0.5672	F=2.163 DFn=5 Dfd=5 P=0.4171	Two-tailed paired t-test	t=1.692 df=5	P=0.1514	ns
Figure S6A	Time in center zone of Open field test (saline vs. CNO circuit activation)	Saline n=6<br>CNO n=7	Mouse	W=0.8649 p=0.2066<br>W=0.8815 p=0.2331	F=1.696 DFn=6 Dfd=5 P=0.5787	Two-tailed unpaired t-test	t=0.1151 df=11	P=0.9104	ns
	Center frequency of Open field test (saline vs. CNO circuit activation)	Saline n=6<br>CNO n=7	Mouse	W=0.9531 p=0.7649<br>W=0.9612 p=0.8288	F=1.088 DFn=6 Dfd=5 P=0.9461	Two-tailed unpaired t-test	t=0.08148 df=11	P=0.9365	ns
	Distance moved of Open field test (saline vs. CNO circuit activation)	Saline n=6<br>CNO n=7	Mouse	W=0.9207 p=0.5105<br>W=0.9316 p=0.5642	F=1.506 DFn=6 Dfd=5 P=0.6697	Two-tailed unpaired t-test	t=0.8599 df=11	P=0.4082	ns
	Velocity of Open field test (saline vs. CNO circuit activation)	Saline n=6<br>CNO n=7	Mouse	W=0.9207 p=0.5107<br>W=0.9318 p=0.5663	F=1.453 DFn=6 Dfd=5 P=0.6985	Two-tailed unpaired t-test	t=0.7120 df=11	P=0.4913	ns
Figure S6B	Time in open arms of Elevated plus maze test (saline vs. CNO circuit activation)	Saline n=6<br>CNO n=7	Mouse	W=0.8489 p=0.1541<br>W=0.8886 p=0.2673	F=1.220 DFn=5 Dfd=6 P=0.8035	Two-tailed unpaired t-test	t=0.2363 df=11	P=0.8176	ns
	Open arm frequency of EPM test (saline vs. CNO circuit activation)	Saline n=6<br>CNO n=7	Mouse	W=0.9563 p=0.7906<br>W=0.8187 p=0.0624	F=1.870 DFn=6 Dfd=5 P=0.5089	Two-tailed unpaired t-test	t=0.4716 df=11	P=0.6464	ns
Figure S6C	Day 2 SI-ratio of EGFP controls (saline vs. CNO)	Saline n=8<br>CNO n=8	Mouse	W=0.8608 p=0.1224<br>W=0.8346 p=0.0663	F=2.114 DFn=7 Dfd=7 P=0.3444	Two-tailed unpaired t-test	t=0.1104 df=14	P=0.9137	ns
	Day 2 Time in SI zone of EGFP controls (saline vs. CNO)	Saline n=8<br>CNO n=8	Mouse	W=0.9587 p=0.7978<br>W=0.8494 p=0.0940	F=1.142 DFn=7 Dfd=7 P=0.8650	Two-tailed unpaired t-test	t=1.610 df=14	P=0.1298	ns
	Day 2 Frequency in SI zone of EGFP controls (saline vs. CNO)	Saline n=8<br>CNO n=8	Mouse	W=0.9670 p=0.8737<br>W=0.9515 p=0.7267	F=3.003 DFn=7 Dfd=7 P=0.1702	Two-tailed unpaired t-test	t=0.4971 df=14	P=0.6268	ns
	Day 2 Time in corner zone of EGFP controls (saline vs. CNO)	Saline n=8<br>CNO n=8	Mouse	W=0.8468 p=0.0885<br>W=0.9337 p=0.5501	F=2.587 DFn=7 Dfd=7 P=0.2330	Two-tailed unpaired t-test	t=0.2910 df=14	P=0.7753	ns
	Day 2 Frequency in corner zone of EGFP controls (saline vs. CNO)	Saline n=8<br>CNO n=8	Mouse	W=0.8926 p=0.2476<br>W=0.9786 p=0.9556	F=4.015 DFn=7 Dfd=7 P=0.0868	Two-tailed unpaired t-test	t=1.544 df=14	P=0.1449	ns
	Day 2 Freezing time of EGFP controls (saline vs. CNO)	Saline n=8<br>CNO n=8	Mouse	W=0.9453 p=0.6644<br>W=0.8361 p=0.0687	F=2.935 DFn=7 Dfd=7 P=0.1788	Two-tailed unpaired t-test	t=1.518 df=14	P=0.1514	ns
Figure S6D	Time in center zone of Open field test (saline vs. CNO circuit inhibition)	Saline n=8<br>CNO n=9	Mouse	W=0.8382 p=0.0722<br>W=0.9720 p=0.9109	F=12.89 DFn=7 Dfd=8 P=0.0017	Two-tailed unpaired t-test, Welch's correction	t=1.111 df=7.965	P=0.2988	ns
	Center frequency of Open field test (saline vs. CNO circuit inhibition)	Saline n=8<br>CNO n=9	Mouse	W=0.9806 p=0.9658<br>W=0.8662 p=0.1119	F=5.170 DFn=7 Dfd=8 P=0.0342	Two-tailed unpaired t-test, Welch's correction	t=1.292 df=9.371	P=0.2273	ns
	Distance moved of Open field test (saline vs. CNO circuit inhibition)	Saline n=8<br>CNO n=9	Mouse	W=0.9461 p=0.6722<br>W=0.9831 p=0.9783	F=1.221 DFn=8 Dfd=7 P=0.8053	Two-tailed unpaired t-test	t=0.9982 df=15	P=0.3340	ns
	Velocity of Open field test (saline vs. CNO circuit inhibition)	Saline n=8<br>CNO n=9	Mouse	W=0.9439 p=0.6496<br>W=0.9839 p=0.9812	F=1.265 DFn=8 Dfd=7 P=0.7696	Two-tailed unpaired t-test	t=0.9677 df=15	P=0.3485	ns
Figure S6E	Time in open arms of Elevated plus maze test (saline vs. CNO circuit inhibition)	Saline n=8<br>CNO n=9	Mouse	W=0.5664 p<0.0001<br>W=0.9336 p=0.5164	F=21.71 DFn=7 Dfd=8 P=0.0003	Mann-Whitney U test	U=34.5	P=0.9081	ns
	Open arm frequency of EPM test (saline vs. CNO circuit inhibition)	Saline n=8<br>CNO n=9	Mouse	W=0.8023 p=0.0303<br>W=0.8828 p=0.1679	F=1.834 DFn=8 Dfd=7 P=0.4388	Mann-Whitney U test	U=28	P=0.4485	ns
Figure S7A	Day 1 5 x ITI freezing ratio (saline vs. CNO and mifepristone CNO before circuit activation)	Saline n=8<br>CNO n=8<br>mifepristone+CNO n=8	Mouse			Two-way ANOVA	Trial × Gene/pharmacology F (8, 84) = 0.7430 P=0.6534<br>Trial F (4, 84) = 16.22 P<0.0001<br>Gene/pharmacology F (2, 21) = 6.361 P=0.0069	Tukey's post hoc test saline vs. CNO P=0.0141<br>saline vs. mifepristone+CNO P=0.0155<br>CNO vs. mifepristone+CNO P=0.9990	*<br>*<br>ns
	Day 1 ITI freezing time (saline vs. CNO and mifepristone CNO before circuit activation)	Saline n=8<br>CNO n=8<br>mifepristone+CNO n=8	Mouse	W=0.8806 p=0.1907<br>W=0.9071 p=0.3344<br>W=0.8476 p=0.0901	F (DFn, DFd)=0.01060 (2, 21) P=0.9895	One-way ANOVA	F (2, 21) = 6.359 P=0.0069	Tukey's post hoc test saline vs. CNO P=0.0141<br>saline vs. mifepristone+CNO P=0.0155<br>CNO vs. mifepristone+CNO P=0.9990	*<br>*<br>ns
Figure S7B	Day 2 5 x ITI freezing ratio (saline vs. CNO and mifepristone CNO before circuit activation)	Saline n=8<br>CNO n=8<br>mifepristone+CNO n=8	Mouse			Two-way ANOVA	Trial × Gene/pharmacology F (8, 84) = 0.7148 P=0.6778<br>Trial F (4, 84) = 6.070 P=0.0002<br>Gene/pharmacology F (2, 21) = 4.406 P=0.0252	Tukey's post hoc test saline vs. CNO P=0.0216<br>saline vs. mifepristone+CNO P=0.5977<br>CNO vs. mifepristone+CNO P=0.1531	*<br>ns<br>ns
	Day 2 ITI freezing time (saline vs. CNO and mifepristone CNO before circuit activation)	Saline n=8<br>CNO n=8<br>mifepristone+CNO n=8	Mouse	W=0.9427 p=0.6376<br>W=0.9266 p=0.4853<br>W=0.9711 p=0.9066	F (DFn, DFd)=0.1079 (2, 21) P=0.8982	One-way ANOVA	F (2, 21) = 4.408 P=0.0252	Tukey's post hoc test saline vs. CNO P=0.0216<br>saline vs. mifepristone+CNO P=0.5983<br>CNO vs. mifepristone+CNO P=0.1527	*<br>ns<br>ns
Figure S7C	Day 9 5 x CS (tone) freezing ratio (saline vs. CNO and mifepristone CNO before circuit activation)	Saline n=8<br>CNO n=8<br>mifepristone+CNO n=8	Mouse			Two-way ANOVA	Trial × Gene/pharmacology F (8, 84) = 1.411 P=0.2039<br>Trial F (4, 84) = 3.594 P=0.0094<br>Gene/pharmacology F (2, 21) = 3.435 P=0.0512	Tukey's post hoc test saline vs. CNO P=0.0484<br>saline vs. mifepristone+CNO P=0.7689<br>CNO vs. mifepristone+CNO P=0.1810	*<br>ns<br>ns
	Day 9 CS (tone) freezing time (saline vs. CNO and mifepristone CNO before circuit activation)	Saline n=8<br>CNO n=8<br>mifepristone+CNO n=8	Mouse	W=0.9548 p=0.7589<br>W=0.7748 p=0.0153<br>W=0.7917 p=0.0233	F (DFn, DFd)=3.696 (2, 21) P=0.0421	Kruskal-Wallis H test	Kruskal-Wallis<br>statistic=7.035<br>p=0.0297	Tukey's post hoc test saline vs. CNO P=0.0441<br>saline vs. mifepristone+CNO P>0.9999<br>CNO vs. mifepristone+CNO P=0.1017	*<br>ns<br>ns
Figure S7D	Day 9 5 x ITI freezing ratio (saline vs. CNO and mifepristone CNO before circuit activation)	Saline n=8<br>CNO n=8<br>mifepristone+CNO n=8	Mouse			Two-way ANOVA	Trial × Gene/pharmacology F (8, 84) = 1.493 P=0.1720<br>Trial F (4, 84) = 1.881 P=0.1213<br>Gene/pharmacology F (2, 21) = 2.072 P=0.1509	Tukey's post hoc test saline vs. CNO P=0.1284<br>saline vs. mifepristone+CNO P=0.5867<br>CNO vs. mifepristone+CNO P=0.5616	ns<br>ns<br>ns
	Day 9 ITI freezing time (saline vs. CNO and mifepristone CNO before circuit activation)	Saline n=8<br>CNO n=8<br>mifepristone+CNO n=8	Mouse	W=0.8953 p=0.2619<br>W=0.9232 p=0.4560<br>W=0.9616 p=0.8256	F (DFn, DFd)=0.7906 (2, 21) P=0.4666	One-way ANOVA	F (2, 21) = 2.074 P=0.1507	Tukey's post hoc test saline vs. CNO P=0.1282<br>saline vs. mifepristone+CNO P=0.5871<br>CNO vs. mifepristone+CNO P=0.5607	ns<br>ns<br>ns
		Saline n=7		W=0.9439 p=0.6741				Tukey's post hoc test Saline vs. Saline SPS&S P=0.0012	**

Figure S7E	Comparison of ITI freezing time on day1 of saline/saline SPS&S/CNO/CNO SPS&S groups	Saline SPS&S n=7<br>CNO n=7<br>CNO SPS&S n=7	Mouse	W=0.9904 p=0.9939<br>W=0.9470 p=0.7022<br>W=0.9391 p=0.6307	F (DFn, DFd)=0.6237 (3, 24) P=0.6066	One-way ANOVA	F (3, 24) = 17.73 P<0.0001	Saline vs. CNO P=0.0002 Saline vs. CNO SPS&S P<0.0001<br>Saline SPS&S vs. CNO P=0.8813 Saline SP&S vs. CNO SPS&S P=0.0558<br>CNO vs. CNO SPS&S P=0.2270	***/****<br>ns/ns<br>ns
Figure S7E	Comparison of ITI freezing time on day2 of saline/saline SPS&S/CNO/CNO SPS&S groups	Saline n=7<br>Saline SPS&S n=7<br>CNO n=7<br>CNO SPS&S n=7	Mouse	W=0.9685 p=0.8875<br>W=0.9832 p=0.9736<br>W=0.9492 p=0.7220<br>W=0.8152 p=0.0577	F (DFn, DFd)=0.7939 (3, 24) P=0.5093	One-way ANOVA	F (3, 24) = 12.43 P<0.0001	Tukey's post hoc test Saline vs. Saline SPS&S P=0.0023<br>Saline vs. CNO P=0.0013 Saline vs. CNO SPS&S P<0.0001<br>Saline SPS&S vs. CNO P=0.9964 Saline SP&S vs. CNO SPS&S P=0.3207<br>CNO vs. CNO SPS&S P=0.4331	**<br>**/****<br>ns/ns<br>ns
Figure S7F	Comparison of CS (tone) freezing time on day9 of saline/saline SPS&S/CNO/CNO SPS&S groups	Saline n=7<br>Saline SPS&S n=7<br>CNO n=7<br>CNO SPS&S n=7	Mouse	W=0.8823 p=0.2368<br>W=0.8627 p=0.1600<br>W=0.5762 p=0.0001<br>W=0.8743 p=0.2021	F (DFn, DFd)=2.241 (3, 24) P=0.1094	Kruskal-Wallis H test	Kruskal-Wallis statistic=12.82 p=0.0050	Dunn post hoc test Saline vs. Saline SPS&S P=0.2341<br>Saline vs. CNO P=0.0167 Saline vs. CNO SPS&S P=0.0082<br>Saline SPS&S vs. CNO >0.9999 Saline SP&S vs. CNO SPS&S >0.9999<br>CNO vs. CNO SPS&S >0.9999	ns<br>*/**<br>ns/ns<br>ns
Figure S7F	Comparison of ITI freezing time on day9 of saline/saline SPS&S/CNO/CNO SPS&S groups	Saline n=7<br>Saline SPS&S n=7<br>CNO n=7<br>CNO SPS&S n=7	Mouse	W=0.9596 p=0.8154<br>W=0.9737 p=0.9238<br>W=0.9567 p=0.7897<br>W=0.9288 p=0.5407	F (DFn, DFd)=0.9175 (3, 24) P=0.4473	One-way ANOVA	F (3, 24) = 7.603 P=0.0010	Tukey's post hoc test Saline vs. Saline SPS&S P=0.1376<br>Saline vs. CNO P=0.0071 Saline vs. CNO SPS&S P=0.0009<br>Saline SPS&S vs. CNO P=0.5364 Saline SP&S vs. CNO SPS&S P=0.1472<br>CNO vs. CNO SPS&S P=0.8257	ns<br>**/***<br>ns/ns<br>ns
Figure S8A	Day 1 habituation freezing ratio (saline vs. CNO EGFP control)	Saline n=8<br>CNO n=8	Mouse	W=0.8421 p=0.0792<br>W=0.9719 p=0.9126	F=4.819 DFn=7 Dfd=7 P=0.0528	Two-tailed unpaired t-test	t=0.02553 df=14	P=0.9800	ns
Figure S8A	Day 1 5 x CS-US freezing ratio (saline vs. CNO EGFP control)	Saline n=8<br>CNO n=8	Mouse			Two-way ANOVA	Trial × Gene/pharmacology F (2.035, 28.49) = 1.424 P=0.2575<br>Trial F (2.035, 28.49) = 17.33 P<0.0001<br>Gene/pharmacology F (1, 14) = 2.209 P=0.1594	Tukey's post hoc test P(trial1 Saline vs. CNO)=0.2652<br>P(trial2 Saline vs. CNO)=0.5939 P(trial3 Saline vs. CNO)=0.1315<br>P(trial4 Saline vs. CNO)=0.1248 P(trial5 Saline vs. CNO)=0.1093	ns
Figure S8A	Day 1 CS (tone) freezing time (saline vs. CNO EGFP control)	Saline n=8<br>CNO n=8	Mouse	W=0.9113 p=0.3634<br>W=0.9769 p=0.9461	F=1.068 DFn=7 Dfd=7 P=0.9332	Two-tailed unpaired t-test	t=1.478 df=14	P=0.1615	*
Figure S8A	Day 1 5 x ITI freezing ratio (saline vs. CNO EGFP control)	Saline n=8<br>CNO n=8	Mouse			Two-way ANOVA	Trial × Gene/pharmacology F (2.527, 35.38) = 0.6369 P=0.5703<br>Trial F (2.527, 35.38) = 6.444 P=0.0022<br>Gene/pharmacology F (1, 14) = 0.2819 P=0.6038	Tukey's post hoc test P(trial1 Saline vs. CNO)=0.1781<br>P(trial2 Saline vs. CNO)=0.5995 P(trial3 Saline vs. CNO)=0.7794<br>P(trial4 Saline vs. CNO)=0.3999 P(trial5 Saline vs. CNO)=0.8095	ns
Figure S8A	Day 1 ITI freezing time (saline vs. CNO EGFP control)	Saline n=8<br>CNO n=8	Mouse	W=0.9566 p=0.7774<br>W=0.8942 p=0.2560	F=3.034 DFn=7 Dfd=7 P=0.1663	Two-tailed unpaired t-test	t=0.5291 df=14	P=0.6051	ns
Figure S8B	Day 2 habituation freezing ratio (saline vs. CNO EGFP control)	Saline n=8<br>CNO n=8	Mouse	W=0.9403 p=0.6144<br>W=0.9475 p=0.6857	F=2.756 DFn=7 Dfd=7 P=0.2044	Two-tailed unpaired t-test	t=0.3498 df=14	P=0.7317	ns
Figure S8B	Day 2 5 x CS (tone) freezing ratio (saline vs. CNO EGFP control)	Saline n=8<br>CNO n=8	Mouse			Two-way ANOVA	Trial × Gene/pharmacology F (1.831, 25.63) = 0.1443 P=0.8488<br>Trial F (1.831, 25.63) = 1.552 P=0.2314<br>Gene/pharmacology F (1, 14) = 1.155 P=0.3006	Tukey's post hoc test P(trial1 Saline vs. CNO)=0.7829<br>P(trial2 Saline vs. CNO)=0.2922 P(trial3 Saline vs. CNO)=0.4472<br>P(trial4 Saline vs. CNO)=0.1877 P(trial5 Saline vs. CNO)=0.6568	ns
Figure S8B	Day 2 CS (tone) freezing time (saline vs. CNO EGFP control)	Saline n=8<br>CNO n=8	Mouse	W=0.9421 p=0.6322<br>W=0.7191 p=0.0037	F=1.151 DFn=7 Dfd=7 P=0.8579	Mann-Whitney U test	U=19.50	P=0.2047	ns
Figure S8B	Day 2 5 x ITI freezing ratio (saline vs. CNO EGFP control)	Saline n=8<br>CNO n=8	Mouse			Two-way ANOVA	Trial × Gene/pharmacology F (2.469, 34.57) = 1.214 P=0.3151<br>Trial F (2.469, 34.57) = 0.9797 P=0.4006<br>Gene/pharmacology F (1, 14) = 1.517 P=0.2384	Tukey's post hoc test P(trial1 Saline vs. CNO)=0.0756<br>P(trial2 Saline vs. CNO)=0.0758 P(trial3 Saline vs. CNO)=0.2077<br>P(trial4 Saline vs. CNO)=0.9186 P(trial5 Saline vs. CNO)=0.8006	ns
Figure S8B	Day 2 ITI freezing time (saline vs. CNO EGFP control)	Saline n=8<br>CNO n=8	Mouse	W=0.9568 p=0.7788<br>W=0.9791 p=0.9582	F=1.915 DFn=7 Dfd=7 P=0.4108	Two-tailed unpaired t-test	t=1.233 df=14	P=0.2378	ns
